# Malectin Alleviates Endoplasmic Reticulum Stress in Gestational Diabetes Mellitus via Glycoprotein Quality Control Mechanisms

**DOI:** 10.1002/advs.202508901

**Published:** 2026-05-25

**Authors:** Jiahui Zhu, Yumeng Zhang, Ye Wang, Xiaoyu Zhu, Ailin Yuan, Weijing Yin, Huangmin Yu, Xuemin Pang, Yufeng He, Yuchen Wang, Tong Wang, Yong Li, Yunlong Si

**Affiliations:** ^1^ Jiangsu Key Laboratory of Brain Disease Bioinformation Research Center for Biochemistry and Molecular Biology Xuzhou Medical University Xuzhou China; ^2^ National Experimental Demonstration Center for Basic Medicine Education Xuzhou Medical University Xuzhou China; ^3^ Xuzhou Maternity and Child Health Care Hospital Xuzhou China

**Keywords:** endoplasmic reticulum stress, gestational diabetes mellitus, glycoprotein quality control, malectin, structure and function

## Abstract

Gestational diabetes mellitus (GDM), is a prevalent metabolic disorder associated with placental dysfunction and adverse pregnancy outcomes. Emerging evidence points to endoplasmic reticulum (ER) stress as a potential initiating factor in GDM pathogenesis. Moreover, ER stress‐mediated trophoblast dysfunction is recognized as a critical pathological mechanism, yet its precise role remains incompletely elucidated. Here, we demonstrate that malectin, an ER‐resident lectin, is upregulated in GDM placentas and protects trophoblasts against high glucose (HG)‐induced ER stress. Mechanistically, malectin recognizes Glc2‐N‐glycans on misfolded glycoproteins via six essential carbohydrate‐binding residues, thereby facilitating glycoprotein quality control (GQC). Malectin knockdown exacerbated HG‐induced ER stress, apoptosis, and impaired trophoblast invasion, syncytialization, and glucose uptake, whereas its overexpression attenuated these defects. Structural analyses revealed the molecular basis for malectin's specificity toward Glc2‐N‐glycan motifs. Importantly, administration of TAT‐Malectin ameliorated hyperglycemia and placental ER stress in a GDM mouse model. In summary, our study provides the first evidence that malectin protects placental trophoblasts from HG‐induced ER stress and damage through GQC mediated by its six key carbohydrate‐binding residues. These findings establish malectin as both a key endogenous placental protector and a promising protein therapeutic candidate, offering a novel target and therapeutic strategy for GDM.

## Graphical abstract

Malectin alleviates high glucose‐induced ER stress and damage in placental trophoblasts, a function dependent on its six critical carbohydrate‐binding residues. In a GDM mouse model, administration of TAT‐Malectin ameliorated hyperglycemia and placental ER stress and prevented fetal macrosomia. This identifies malectin as a key placental protector and a promising protein therapeutic candidate for GDM.

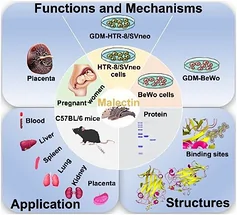

## 1 Introduction

Gestational diabetes mellitus (GDM), one of the most prevalent metabolic complications during pregnancy, is defined as glucose intolerance that develops or is first recognized during gestation. It increases the risk of maternal complications such as preeclampsia, macrosomia, and stillbirth [1, 2]. The global prevalence of GDM continues to rise, with statistical data from 2024 indicating a prevalence of approximately 14%, making it a significant global public health concern [3]. With the sharp increase in the number of pregnancies of elderly pregnant women, the incidence of GDM has further risen, causing a huge health and economic burden. Clinically, GDM management is primarily reliant on insulin therapy. However, this approach may induce adverse effects in mothers, including weight gain, hypoglycemia, and allergic reactions, as well as neonatal complications such as hypoglycemia and respiratory distress syndrome [4]. Traditional research on GDM has focused on insulin signaling pathways and inflammatory cytokines. With advancements in high‐throughput sequencing and multi‐omics technologies, studies leveraging metabolomics and gut microbiota analysis have emerged to explore the underlying mechanisms of GDM pathogenesis [5, 6]. Despite these efforts, the precise pathogenesis of GDM remains incompletely understood, underscoring the urgent need to identify novel pathological mechanisms and develop innovative therapeutic strategies.

The endoplasmic reticulum (ER) is a critical organelle for protein synthesis, post‐translational modification, and processing. Endogenous and exogenous cellular stressors, including high glucose, viral infection, inflammation, and environmental toxins, can induce the accumulation of unfolded or misfolded proteins in the ER, triggering ER stress and unfolded protein response (UPR) [7, 8]. During the compensatory phase, ER stress restores proteostasis; however, persistent stimuli activate mitochondrial‐dependent apoptosis via core regulatory molecules such as C/EBP homologous protein (CHOP), which also mediates inflammatory response imbalance and autophagy dysregulation [7, 9]. Mounting evidence underscores the critical role of ER stress and the associated UPR signaling pathways in the normal physiological function of reproductive tissues. These processes are integral to endometrial changes during the menstrual cycle, placental development, and the maintenance of pregnancy, as well as the initiation of parturition. Conversely, the disruption of ER homeostasis, which results from an excessive accumulation of unfolded/misfolded proteins due to prolonged or severe ER stress, can trigger various pathological conditions. Such dysregulation has been implicated in the pathogenesis of endometriosis, endometrial/ovarian cancers, and a spectrum of pregnancy complications [10, 11, 12]. These complications include preeclampsia (PE), spontaneous abortion (SA), recurrent pregnancy loss (RPL), macrosomia, intrauterine growth restriction (IUGR), and preterm birth (PTB) [13, 14, 15, 16, 17]. Notably, these adverse pregnancy outcomes are frequently characterized by excessive elevation of ER stress, which appears to play a pivotal role in their underlying mechanisms. Consequently, targeting ER stress presents a promising avenue for novel therapeutic interventions for these gestational diseases. Histological studies by Graham J. Burton et al. first demonstrated aberrant expression of ER stress markers (e.g., GRP78, XBP1) in GDM placentas [18], and Feng Ling et al. further elucidated the NF‐κB/IL‐6 axis‐mediated exacerbation of oxidative damage and inflammatory cascades in placental tissues under ER stress [19]. Although traditional studies have focused on abnormal insulin signaling pathways and imbalances of inflammatory factors, emerging evidence suggests that ER stress activation in trophoblasts occurs earlier than systemic insulin resistance, potentially serving as an initiating factor in GDM pathogenesis [20]. Husamettin Vatansev et al. identified ATF‐6 as a potential biomarker for GDM, and its levels were significantly correlated with GDM and key clinical parameters [21]. These findings support the inclusion of ER stress in future research and clinical practice regarding GDM. Therefore, targeted inhibition of ER stress to alleviate trophoblast cell injury induced by high glucose may provide a novel intervention strategy for the treatment of GDM.

In mammalian systems, the majority of proteins synthesized by the rough endoplasmic reticulum are modified with *N*‐linked glycans, which are critical for proper protein maturation. These glycans function as key recognition signals within the glycoprotein quality control (GQC) system [22]. The interplay between the GQC machinery and the ER stress response mechanisms, such as UPR, is fundamental to cellular homeostasis. This coordination is indispensable for normal cellular differentiation, growth, tissue morphogenesis, and embryonic development. Defects in this system underlie several pathologies, including congenital disorders of glycosylation (CDGs), α1‐antitrypsin (α1AT) deficiency, and combined factor V and factor VIII deficiency (F5F8D) [23]. Furthermore, GQC dysregulation is closely associated with diabetes mellitus, including gestational diabetes (GDM). Evidence indicates that alterations in the *N*‐glycome are present in individuals with type 2 diabetes, and specific *N*‐glycan profiles may even identify those at elevated risk for developing the disease [24]. Supporting this, genome‐wide association studies have implicated glycosyltransferase genes as potential causal factors in both type 1 and type 2 diabetes [25]. Additionally, *N*‐glycans are proposed to play a significant role in preserving glucose‐stimulated insulin secretion, potentially through the modulation of glucose transporter expression on the cell surface [26]. In GDM, *N*‐glycosylation abnormalities have been reported, notably glycosylation defects in glycodelin‐A—a decidual glycoprotein with immunomodulatory functions dependent on glycan moieties [27]. Recent advances in mass spectrometry‐based proteomics have also identified several dysregulated glycoproteins in GDM that modulate insulin signaling pathways either directly or indirectly [28]. Based on the established role of GQC in maintaining proteostasis and the accumulating evidence of its dysregulation—as reflected by altered N‐glycan profiles, implicated glycosyltransferase genes, and specific glycoprotein defects affecting insulin signaling and placental function—targeting the GQC may serve as a promising therapeutic strategy for restoring metabolic homeostasis in GDM.

Malectin is an ER‐resident lectin initially identified in the African clawed frog (*Xenopus laevis*) in 2008 [29]. This protein was termed “malectin” following the discovery that its first identified disaccharide ligand was maltose. Subsequent studies revealed that after the outermost glucose residue of the Glc_3_‐Man_9_‐GlcNAc_2_ (Glc3‐N‐glycan) oligosaccharide is cleaved by glucosidase I, the resulting Glc_2_‐Man_9_‐GlcNAc_2_ (Glc2‐N‐glycan) structure is specifically recognized and bound by Malectin with a dissociation constant (Kd) of 1.97 × 10^5^ m ^−^^1^ [30]. Correctly folded proteins undergo further processing by α‐glucosidase II, which excises the second glucose residue to yield Glc_1_Man_9_GlcNAc_2_ (Glc1‐N‐glycan). This intermediate is recognized by the calnexin/calreticulin (CNX/CRT) chaperone system to complete quality control. Misfolded proteins, however, retain partial Glc2‐N‐glycan structures, which are specifically recognized by malectin as molecular tags [30]. Malectin binds Glc2‐N‐glycans via its carbohydrate recognition domain (CRD), forming a complex with ribosome‐associated protein I (RPN1) to retain misfolded glycoproteins in the ER lumen and prevent their vesicular transport to the Golgi [31]. Currently, no studies have investigated the role of malectin in the placenta or in pregnancy‐related disorders. However, emerging evidence indicates its involvement in other pathological conditions. Proteomic profiling of thyroid papillary carcinoma has identified malectin as a potential biomarker for the disease [32]. Additionally, malectin expression is elevated in patients with atopic dermatitis [33]. In hepatocellular carcinoma cells, knockdown of malectin suppresses cell migration and invasion capabilities [34]. Functional studies demonstrate that malectin facilitates the synthesis and secretion of normal coagulation factor VIII and assists in the refolding of the mutant VIII D1241E, thereby reducing its secretion—highlighting its critical role in protein quality control [35]. Furthermore, it participates in inflammatory responses by modulating the polarization of M1/M2 macrophages [36]. Notably, the synergistic interplay between chronic inflammation and ER stress has been established as a core pathological feature of GDM [20]. Therefore, we speculate that malectin alleviates ER stress via GQC in GDM.

In this study, we demonstrate that malectin alleviates high glucose‐induced ER stress and damage in placental trophoblasts, a function dependent on its six critical carbohydrate‐binding residues. Intraperitoneal administration of TAT‐Malectin to GDM mice improves glucose metabolism. This identifies malectin as a key placental protector and a promising protein therapeutic candidate for GDM. In summary, our study provides the first evidence linking malectin to the molecular pathogenesis of GDM, offering a novel therapeutic target and potential new directions for GDM treatment.

## 2 Results

### 2.1 ER Stress and Malectin Expression Levels are Significantly Upregulated in Placental Tissues From Women With GDM

**FIGURE 1:**
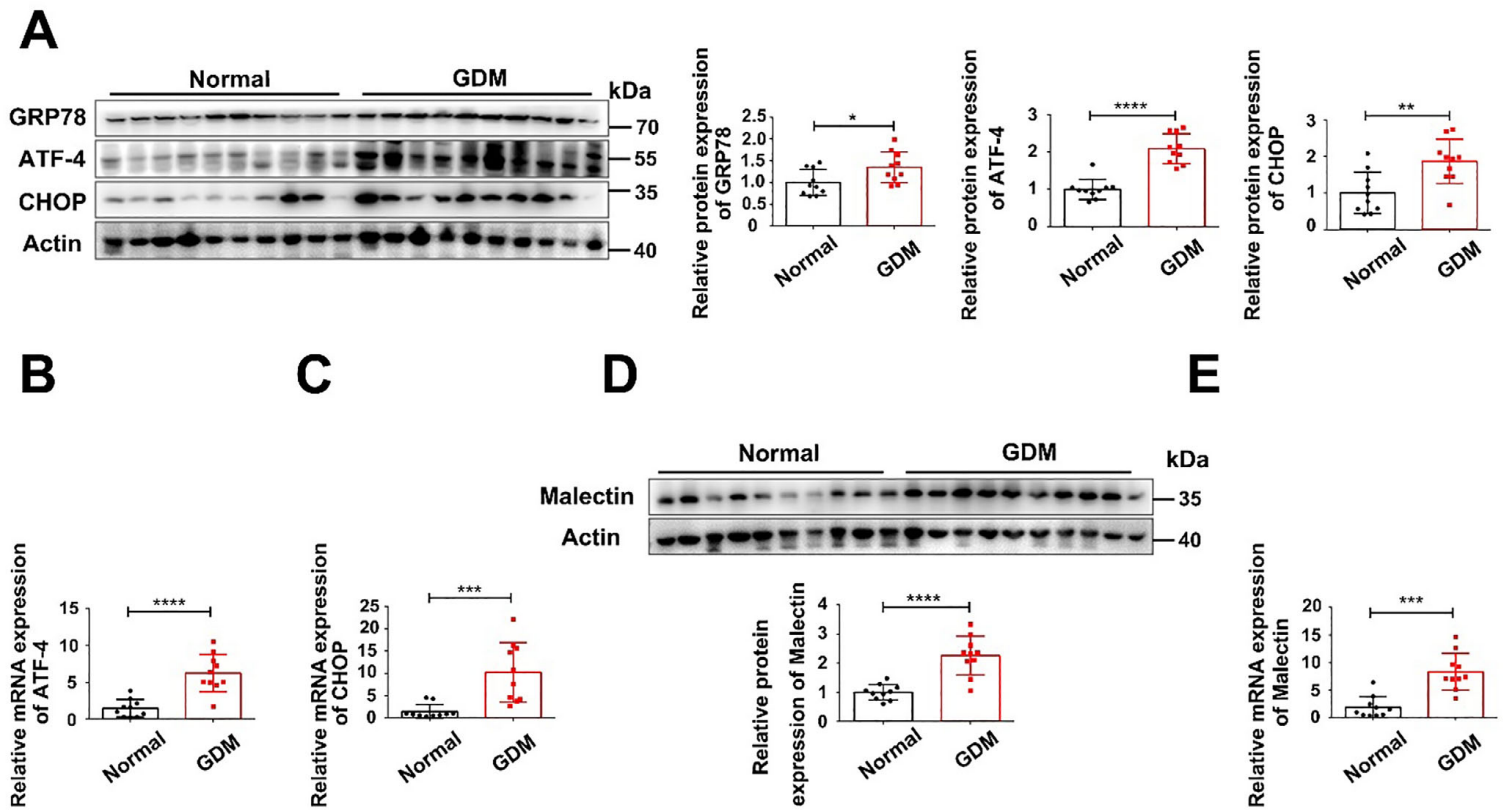
Malectin expression is elevated in GDM placentas and associated with ER stress. (A–C) Protein and mRNA expression levels of the ER stress markers GRP78, ATF‐4, and CHOP in placental tissues from control and GDM pregnancies were determined by Western blot and qPCR. For Western blot quantification, band intensities were normalized to Actin. (D,E) Protein and mRNA expression levels of malectin were determined by Western blot (normalized to Actin) and qPCR. Data are presented as mean ± SD (n = 10 biologically independent samples per group). Statistical significance was determined by unpaired two‐tailed Student's t‐test (^****^ *p* < 0.0001, ^***^ *p* < 0.001, ^**^ *p* < 0.01 vs. control group).

Table S1 presents clinical characteristics of the pregnancies without or with GDM (Normal and GDM). Maternal second‐trimester BMI differed significantly between normal and GDM groups (24.45 ± 1.119 vs. 28.28 ± 3.821, *p* = 0.0071), whereas no difference was observed in third‐trimester BMI. This may be due to the restriction of carbohydrate and fat intake in the third trimester with GDM, and the calorie supply was closer to the fetal demand rather than maternal storage, resulting in an increase of BMI similar to that of normal pregnant women. No significant differences were observed in maternal age, gestational age, or neonatal birth weight between groups. However, glucose metabolism parameters exhibited marked disparities: fasting blood glucose (4.446 ± 0.3273 vs. 5.353 ± 0.6871, *p* = 0.0014), 1 h post‐OGTT (7.36 ± 1.123 vs. 10.03 ± 1.598, *p* = 0.0004), and 2 h post‐OGTT (6.02 ± 1.142 vs. 9.36 ± 1.563, *p* < 0.0001) values were significantly elevated in GDM pregnancies. To elucidate the pathogenesis of GDM, placental tissues from normal and GDM pregnancies were collected. ER stress has been implicated in GDM progression, with previous studies reporting its regulatory role in metabolic disorders. Quantitative analysis by Western blot and RT‐PCR revealed significant upregulation of ER stress biomarkers in GDM placentas compared to normal controls. At the protein level, expressions of GRP78, ATF‐4, and CHOP were elevated to 1.35‐, 2.09‐, and 1.87‐fold, respectively. Consistent with this, mRNA levels of ATF‐4 and CHOP were increased to 4.3‐ and 7.0‐fold in GDM placentas (Figure 1A–C). These findings align with prior reports by Graham J. Burton and Ling Feng [18, 19], confirming the hyperactivation of ER stress in the pathophysiology of GDM. Furthermore, quantitative analysis revealed that both protein and mRNA levels of malectin were significantly upregulated in GDM placental tissues compared to the normal group (2.26 ± 0.67 vs. 1.0 ± 0.27 and 8.32 ± 3.35 vs. 1.86 ± 1.94, respectively) (Figure 1D,E). This observation suggests that malectin may be involved in the pathological process of GDM.

### 2.2 Malectin Knockdown Disrupts Protein Homeostasis in HTR‐8/SVneo Cells, Leading to ER Stress and Apoptosis

**FIGURE 2:**
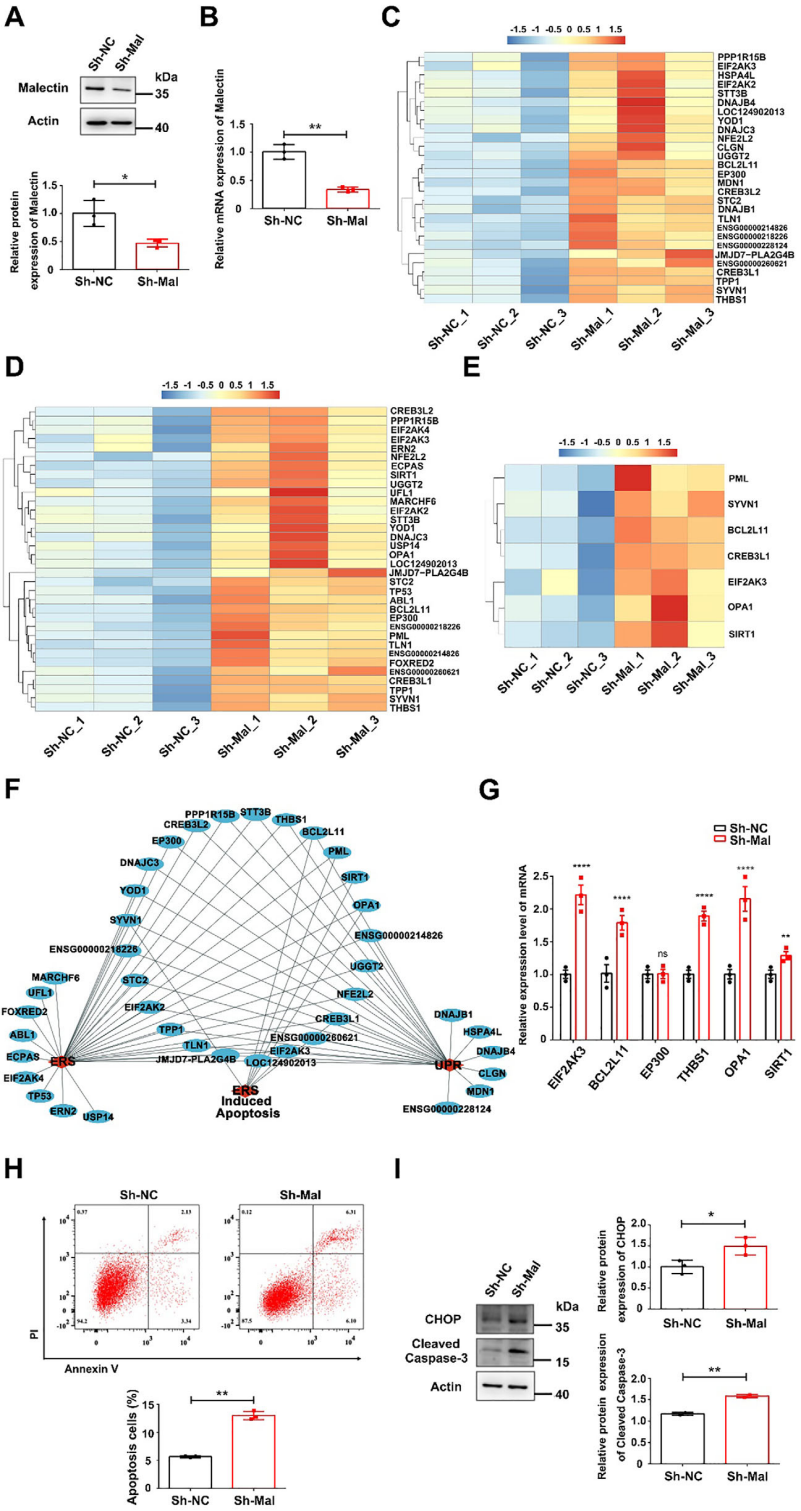
Malectin knockdown induces apoptosis and transcriptomic alterations in HTR‐8/SVneo cells. (A,B) Knockdown efficiency was validated by Western blot with band intensities normalized to Actin and qPCR with expression normalized to Actin (C–E) Heatmaps depicting upregulated genes associated with UPR, ER stress, and ER stress‐induced apoptosis following malectin knockdown. (F) Network diagram showing the interrelationships of the genes among UPR, ER stress and ER stress‐induced apoptosis in cells. (G) qPCR validation of six differentially expressed genes from RNA‐seq data (normalized to Actin). (H) Representative flow cytometry detection of cell apoptosis by Sh‐NC and Sh‐Mal. (I) Western blot analysis of CHOP and cleaved caspase‐3 protein levels (normalized to Actin). Data represent mean ± SD of n = 3 biologically independent experiments. Statistical significance was determined by unpaired two‐tailed Student's t‐test (^****^ *p* < 0.0001, ^***^ *p* < 0.001, ^**^ *p* < 0.01, ^*^ *p* < 0.05, ns > 0.05) with comparisons to Sh‐NC group.

To investigate the functional role of malectin, HTR‐8/SVneo cells were transduced with lentiviral vectors encoding either a malectin‐targeting shRNA (Sh‐Mal) or a non‐targeting control shRNA (Sh‐NC). Knockdown efficiency was confirmed by qPCR and Western blot analysis, showing that malectin mRNA levels were reduced to approximately one‐third, and protein levels were decreased to less than half compared to the Sh‐NC group (Figure 2A,B). RNA‐seq was performed to profile transcriptomic changes. Quality assessment confirmed ≥99% of bases with Q30 scores across all samples, ensuring high‐quality mRNA‐seq data. Given malectin's ER localization and its reported role in protein quality control, we focused on ER‐associated pathways. RNA‐seq revealed that malectin knockdown significantly upregulated genes involved in the UPR, ER stress signaling, and apoptosis. Heatmap visualization demonstrated distinct transcriptomic profiles between Sh‐Mal and Sh‐NC groups (Figure 2C–E). ER stress is a cellular stress response caused by the accumulation of unfolded proteins in the lumen. In response to ER stress, cells have developed an evolutionarily conserved mechanism, known as the UPR. Under homeostatic conditions, UPR activation initiates adaptive responses; however, when stress exceeds the adaptive capacity of the UPR, it triggers apoptosis. The network diagram was used to illustrate the UPR‐ER stress‐apoptosis axis following malectin knockdown (Figure 2F). Subsequently, eight genes, including CREB3L1, EIF2AK3, SYVN1, BCL2L11, EP300, THBS1, SIRT1, and OPA1, were randomly screened from the up‐regulated genes detected by RNA‐seq for qPCR verification. qPCR validation showed no significant difference in EP300 expression between Sh‐Mal and Sh‐NC cells. The expression trends of the other major differentially expressed genes were consistent with the RNA‐seq data, confirming transcriptomic reliability (Figure 2G). In addition, flow cytometry revealed elevated apoptotic rates in Sh‐Mal cells compared to Sh‐NC controls (13.01% ± 0.43% vs. 5.69% ± 0.11%, *p* = 0.0021). Western blot analysis further demonstrated that malectin knockdown elevated the protein levels of cleaved caspase‐3 and CHOP by approximately 1.36‐fold (1.587 ± 0.025 vs. 1.170 ± 0.026, *p* = 0.0075) and 1.49‐fold (1.491 ± 0.120 vs. 1.000 ± 0.093, *p* = 0.0322), respectively (Figure 2H,I). These results showed that malectin knockdown increased apoptosis of HTR‐8/SVneo cells, which was consistent with the previous sequencing results. Based on these findings, we postulate that elevated malectin expression in placental tissues of GDM may represent a homeostatic adaptive mechanism. Upregulation of malectin could modulate UPR signaling to attenuate ER stress and apoptotic cascades, thereby mitigating placental trophoblast damage and exerting cytoprotective effects.

### 2.3 Malectin Knockdown Exacerbates High Glucose (HG)‐Induced Apoptosis and ER Stress While Attenuating Invasion in HTR‐8/SVneo Cells

**FIGURE 3:**
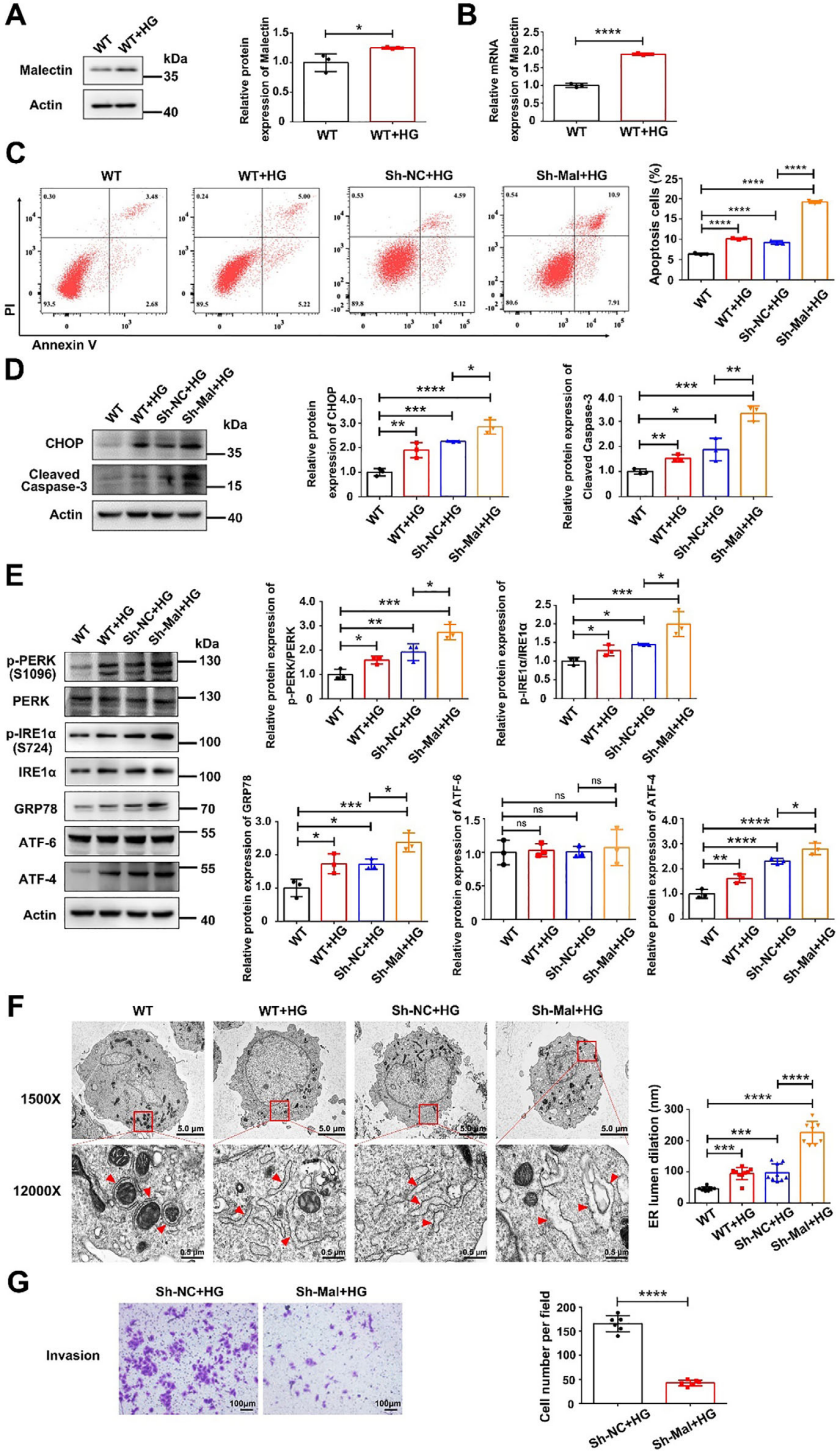
Malectin knockdown exacerbates HG‐induced ER stress and apoptosis while impairing cell invasion in HTR‐8/SVneo cells. (A,B) Malectin expression levels in WT and WT+HG cells were determined by Western blot (band intensities normalized to Actin; n = 3) and qPCR (expression normalized to Actin; n = 3). (C) Apoptosis was assessed by flow cytometry after Annexin V/PI staining (n = 3). The gating strategy involved sequential selection of intact cells based on FSC‐A/SSC‐A, exclusion of doublets using FSC‐H/FSC‐A, and quantification of apoptotic populations (Annexin V+/PI‐ for early apoptosis; Annexin V+/PI+ for late apoptosis/necrosis). (D) Protein levels of CHOP and cleaved caspase‐3 were analyzed by Western blot (normalized to Actin; n = 3). (E) Activation of ER stress pathways was evaluated by Western blot (normalized to Actin; n = 3). (F) ER ultrastructure was examined by TEM; red arrows indicate dilated ER lumen, with quantitative analysis of lumen diameter (n = 9 independent measurements per group). (G) Cell invasion capacity was measured by the Transwell assay. Cell counts were conducted in five independent fields of view per group (n = 5). All quantitative data are presented as mean ± SD. Statistical significance was determined by unpaired two‐tailed Student's t‐test or one‐way ANOVA with Tukey's post hoc test (^****^ *p* < 0.0001, ^***^ *p* < 0.001, ^**^ *p* < 0.01, ^*^ *p* < 0.05, ns > 0.05).

To elucidate the role of malectin in GDM progression, we employed two complementary trophoblast models: HTR‐8/SVneo cells, used to study invasion and apoptosis, and BeWo cells, employed to investigate syncytialization and nutrient transport under HG conditions. Our glucose gradient experiment showed that 20 and 25 mm glucose induced comparable effects on PI3K/AKT/GLUT4 expression, apoptosis, and Malectin levels in HTR‐8/SVneo cells (Figure S2A–C). Based on this result and consistency with numerous established studies, subsequent experiments defined 25 mm glucose as the HG condition. Malectin expression was analyzed in HTR‐8/SVneo cells (wild‐type, WT) and HG‐treated WT cells (WT+HG). Malectin expression was significantly induced in WT+HG cells, with protein and mRNA levels increasing by approximately 1.4‐fold and 2.0‐fold, respectively, compared to controls, aligning with the elevated malectin expression observed in placental tissues of GDM patients (Figure 3A,B). Apoptosis rates were quantified by flow cytometry in four groups: WT, WT+HG, Sh‐NC+HG, and Sh‐Mal+HG. HG increased apoptosis from WT to WT+HG (6.37% ± 0.13% vs. 10.14% ± 0.11%, *p* < 0.0001). Sh‐NC+HG cells exhibited similar apoptosis rates to WT+HG, whereas Sh‐Mal+HG cells showed a nearly two‐fold increase (9.19% ± 0.26% vs. 19.22% ± 0.21%, *p* < 0.0001) (Figure 3C). Western blot analysis of cleaved caspase‐3 and CHOP confirmed these findings (Figure 3D), demonstrating that malectin knockdown amplifies apoptosis in HG‐exposed trophoblasts. Previous RNAseq results showed that malectin knockdown increased UPR and ER stress. UPR activation involves three main signaling pathways, namely IRE1 pathway, PERK pathway, and ATF‐6 pathway. To determine whether malectin knockdown exacerbates ER stress and apoptosis through specific pathways, we first verified the efficacy of our antibodies against key ER stress markers (e.g., PERK, IRE1α, and ATF‐6) via Western blot using tunicamycin‐treated cells (Figure S2D). Subsequently, we probed the activation of these pathways in Sh‐Mal+HG cells. Our results indicated that the ratios of p‑PERK/PERK and p‐IRE1α/IRE1α were significantly increased in WT+HG cells compared with WT cells. In addition, the ratios of Sh‐Mal+HG cells were significantly higher than Sh‐NC+HG cells. Furthermore, GRP78 and ATF4 levels were increased in WT+HG cells compared with WT cells, and these levels of Sh‐Mal+HG cells were significantly higher than Sh‐NC+HG cells, too. However, for ATF‐6 levels, there was no significant difference in expression levels between WT, WT+HG, Sh‐NC+HG, and Sh‐Mal+HG cells (Figure 3E). Using transmission electron microscopy (TEM), we systematically quantified ER lumen dilation—a morphological indicator of ER stress—across the four experimental groups (WT, WT+HG, Sh‐NC+HG, Sh‐Mal+HG). TEM revealed that the ER lumen diameter in the WT group measured approximately 45.76 ± 6.01 nm. In contrast, WT+HG cells exhibited significant ER swelling, with a lumen diameter of 94.54 ± 19.9 nm, representing approximately twofold enlargement compared to the WT group. Sh‐NC+HG cells displayed comparable ER lumen dilation (97.24 ± 27.82 nm) to the WT+HG group. Strikingly, Sh‐Mal+HG cells demonstrated the most severe ER lumen dilation (226.2 ± 35.65 nm), which was approximately twofold greater than that in Sh‐NC+HG cells These findings collectively demonstrated that HG induces ER stress in HTR‐8/SVneo cells, while malectin knockdown exacerbates HG‐triggered ER stress (Figure 3F). Furthermore, Malectin knockdown markedly reduced cell invasion by approximately threefold in HG condition (Figure 3G). To rigorously rule out the possibility of off‐target effects, we designed and validated ​​a second, independent shRNA targeting a distinct sequence of the MLEC gene (Sh‐Mal‐2). We then performed the key functional assays under HG conditions. Strikingly, cells recapitulated all the phenotypic changes observed with the first shRNA (Figure S3). These findings suggest that Malectin deficiency aggravates HG‐induced ER stress primarily through the IRE1α and PERK pathways, leading to increased apoptosis and impaired invasion.

### 2.4 Malectin Knockdown Exacerbates HG‐Induced ER Stress While Attenuating Syncytialization and Glucose Uptake in BeWo Cells

**FIGURE 4:**
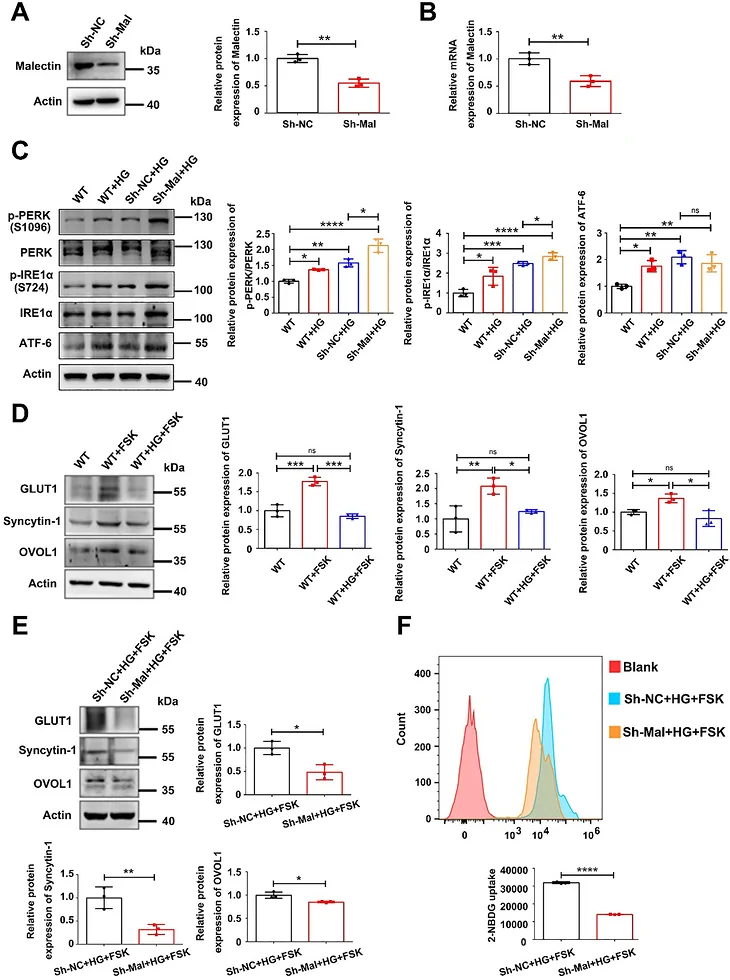
Malectin knockdown exacerbates HG‐induced ER stress while attenuating syncytialization and glucose uptake in BeWo cells. (A,B) Knockdown efficiency was validated by Western blot with band intensities normalized to Actin and qPCR with expression normalized to Actin. (C) Activation of ER stress pathways was evaluated by Western blot (normalized to Actin). (D,E) Western blot analysis of syncytialization and GLUT1 expression levels (normalized to Actin). (F) Glucose uptake was measured by flow cytometry using 2‐NBDG. All quantitative data are presented as mean ± SD from n = 3 biologically independent experiments. Statistical significance was determined by unpaired two‐tailed Student's t‐test or one‐way ANOVA with Tukey's post hoc test (^****^ *p* < 0.0001, ^***^ *p* < 0.001, ^**^ *p* < 0.01, ^*^ *p* < 0.05, ns > 0.05).

To study the functional role of malectin in BeWo cells, cells were also transduced with lentiviral vectors. The lentiviral vectors encoded a malectin‐targeted shRNA (Sh‐Mal) or a non‐targeted control shRNA (Sh‐NC). Western blot and qPCR analyses confirmed the knockdown efficiency, showing that malectin protein and mRNA levels were reduced by approximately 50% in the Sh‐Mal group compared to the Sh‐NC control (Figure 4A,B). HG induced ER stress in BeWo cells, activating all three UPR pathways (IRE1, PERK, and ATF6). Furthermore, Malectin knockdown significantly augmented this response, as evidenced by increased ratios of p‐PERK/PERK and p‐IRE1α/IRE1α in Sh‐Mal+HG cells compared to Sh‐NC+HG controls. In contrast, ATF6 levels remained unchanged upon Malectin knockdown, a result consistent with our prior observations in HTR‐8/SVneo cells. The results indicate that Malectin primarily modulates ER stress through the IRE1 and PERK pathways (Figure 4C). FSK treatment effectively induced syncytialization in BeWo cells, as evidenced by significantly elevated expression of the syncytialization markers Syncytin‐1 (Syn‐1) and OVOL‐1 in the WT+FSK group compared to the WT control. However, HG inhibited this process, reducing Syn‐1 and OVOL‐1 expression. Notably, GLUT1, a key glucose transporter widely expressed in placental cells and reported to promote syncytialization via enhanced glucose uptake in BeWo cells [37]. Our results revealed a significant positive correlation between GLUT1 expression levels and the extent of syncytialization (Figure 4D). Under HG+FSK conditions, Malectin knockdown (Sh‐Mal) in BeWo cells significantly reduced the protein levels of Syn‐1, OVOL‐1, and GLUT1 to 0.3‐fold, 0.8‐fold, and 0.48‐fold, respectively, compared to the Sh‐NC group. Consistent with the downregulation of GLUT1, glucose uptake capacity was also impaired, decreasing to 0.44‐fold (Figure 4E,F). These results suggest that Malectin knockdown may attenuate syncytialization and glucose uptake under HG conditions by downregulating GLUT1 expression.

### 2.5 Mal_42‐228 Exhibits Carbohydrate‐Binding Activities

**FIGURE 5:**
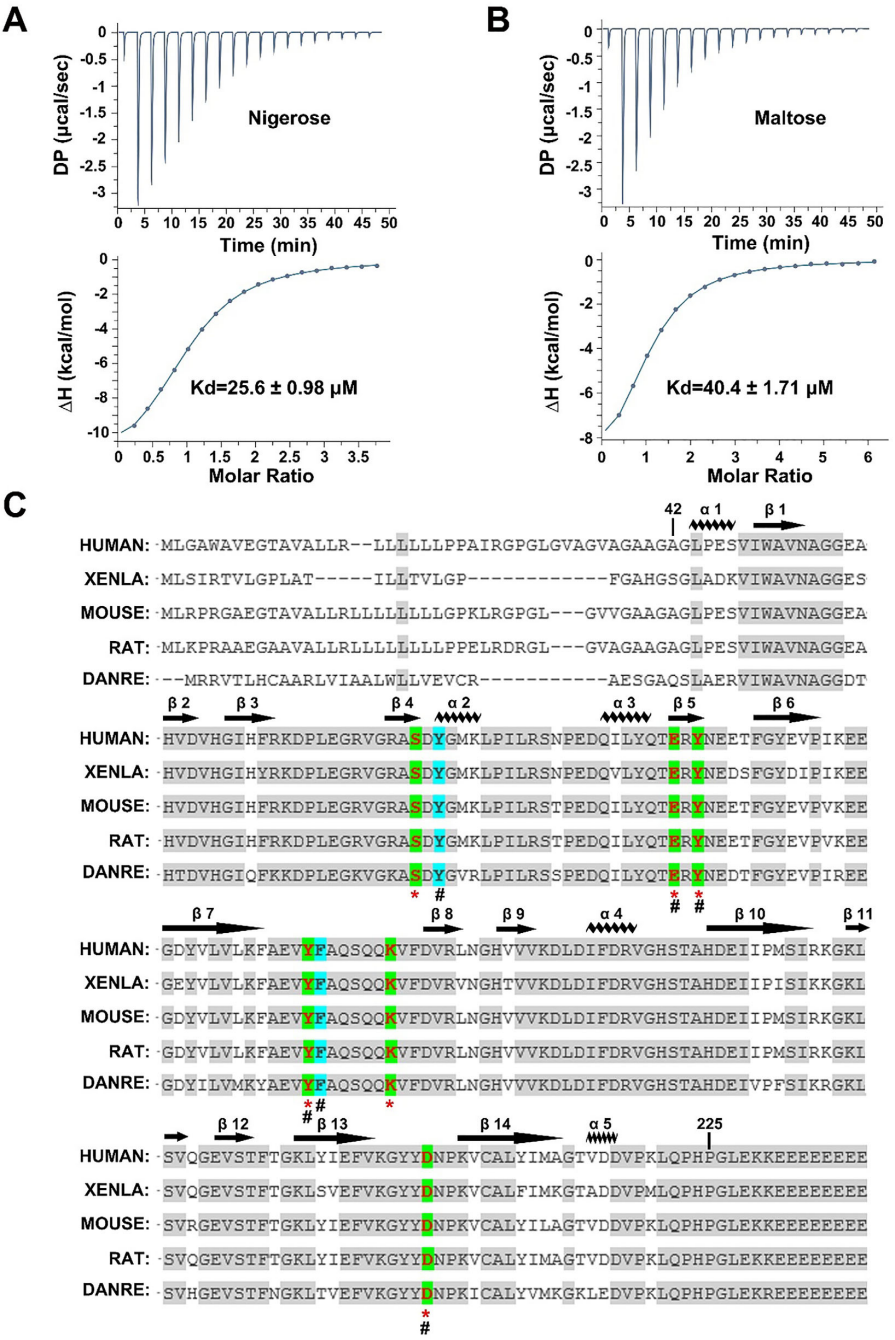
Functional characterization of human malectin: carbohydrate‐binding activity and evolutionary conservation. (A,B) ITC analysis of the binding of Malectin with nigerose and maltose. In each figure, the raw data are shown in the upper panel, and the integrated heat data, corrected for the heat of dilution, are shown in the lower panel. (C) Comparison of homology between human malectin sequences and Xenopus laevis, mouse, rat, and zebrafish. The red ‘^*^’ represents the six carbohydrate‐binding sites of human malectin based on the human malectin structure data determined in this study, while black ‘#’ represents the six carbohydrate‐binding sites of Xenopus laevis malectin based on the previously reported structure data.

To elucidate the molecular mechanism of malectin in ER protein quality control, we employed crystallographic analysis at atomic resolution. First, Mal_42‐228, encompassing the intact CRD of malectin, was heterologously expressed and purified. To verify the biological activity of purified Mal_42‐228, we performed isothermal titration calorimetry (ITC) assay and carbohydrate‐binding activity assay. The purified human malectin protein exhibited binding affinity for the nigerose(Glcα1‐3Glc) and maltose(Glcα1‐4Glc), with dissociation constants (Kd) of 25.6 ± 0.98 εM  and 40.4 ± 1.71 µm, respectively, as determined by ITC. The affinity for nigerose was approximately two‐fold higher than that for maltose (Figure 5A,B). This result is consistent with the previously reported affinities of *Xenopus laevis* malectin, which were 26.3 ± 0.7 µm for nigerose and 50 ± 0.5 µm for maltose [29]. These data confirm that the recombinantly expressed human malectin is correctly folded and retains its specific carbohydrate‐binding activity in vitro. Carbohydrate‐binding activity assay also confirmed Mal_42‐228's preferential interaction with glucose‐, maltose‐, and nigerose‐conjugated Sepharose 6B matrices, whereas mannose‐conjugated and control Sepharose matrices showed no binding (Figure S5A). These results indicate that Mal_42‐228 has a good carbohydrate‐binding activity; meanwhile, glucose, maltose, and nigerose can bind Mal_42‐228. These results provide a good basis for the subsequent crystallography study. In addition, Malectin exhibits high sequence conservation across vertebrate species, with human malectin sharing 83.7%, 95.16%, 95.85%, and 75.63% identity with Xenopus laevis, mouse, rat, and zebrafish orthologs, respectively (Figure 5C). The high sequence conservation of malectin suggests strong functional conservation and underscores the importance of this protein.

### 2.6 Crystal Structures of Mal_42‐228

**FIGURE 6:**
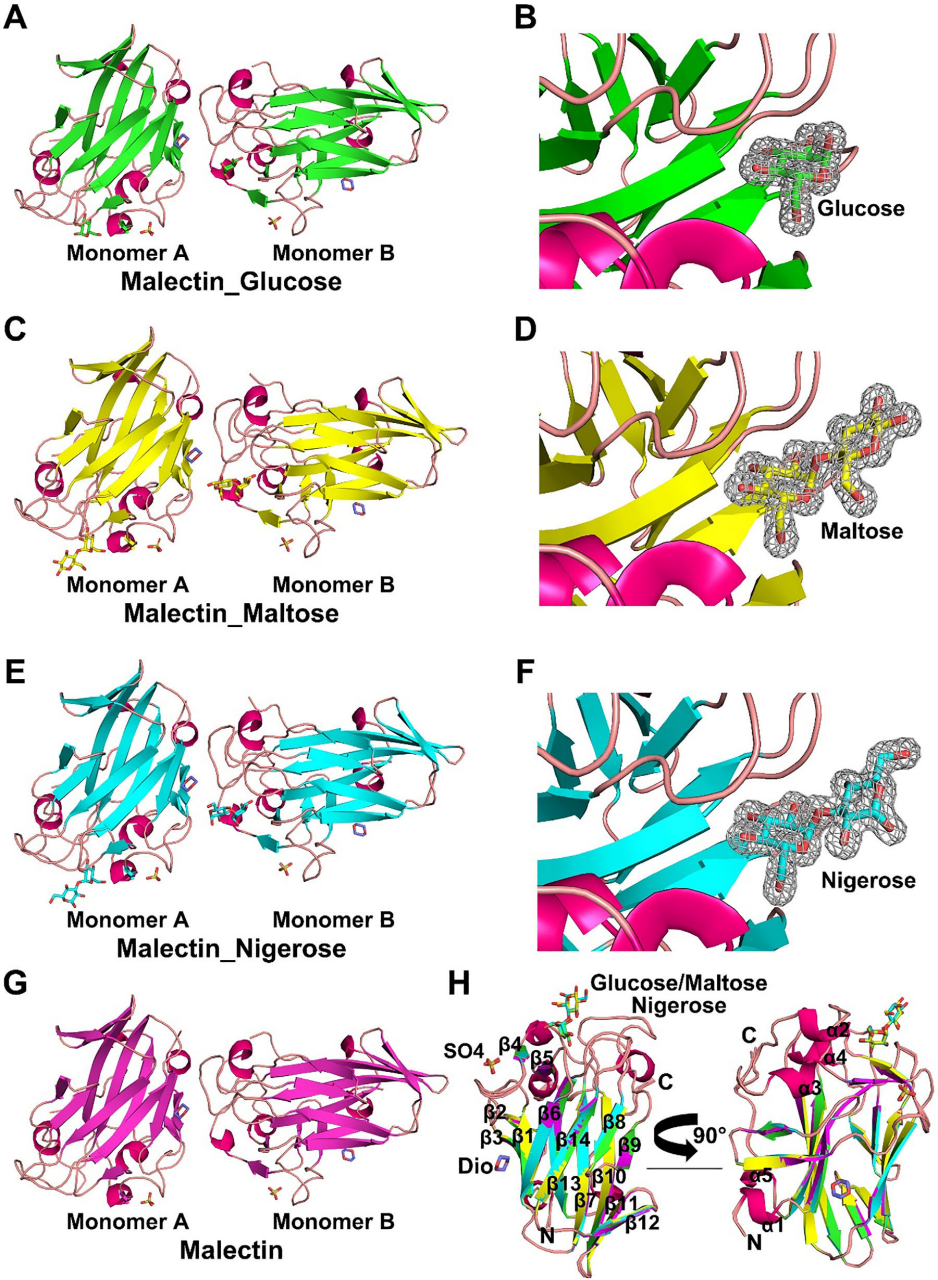
The crystal structure of human malectin. (A,B) The crystal structure of human malectin in complex with glucose (green sheet, PDB ID: 9IL3). (C,D) The crystal structure of human malectin in complex with maltose (yellow sheet, PDB ID: 9ILA). (E,F) The crystal structure of human malectin in complex with nigerose (cyan sheet, PDB ID: 9ILF). (G) The crystal structure of human malectin without carbohydrate (magenta sheet, PDB ID: 9IKP). (H) Alignment of monomer structures of four human malectin above. The secondary structure elements (β‐strands β1–β14 and α‐helices α1–α5) are labeled. The 2|Fo|‐|Fc| map contoured at 1σ, covering all atoms of the carbohydrates was shown.

The structure of human malectin and its carbohydrate recognition mechanism remain poorly characterized. Here, we report the crystal structures of human malectin (PDB ID: 9IKP) and its complexes with glucose (9IL3), maltose (9ILA), and nigerose (9ILF) at resolutions of 1.68, 1.45, 1.56, and 1.56 Å, respectively (Figure 6A–G). All four structures adopt the P31 space group, with structural statistics detailed in Table S4. Structurally, malectin forms a dimeric assembly in all crystal forms, with each monomer exhibiting a canonical β‐sandwich fold comprising 14 β‐strands, 5 α‐helices, and flexible loops. This architecture closely resembles the conserved β‐sandwich motif observed in galectin family proteins. Notably, 1,4‐dioxane molecules, present in the crystallization buffer, co‐crystallized with malectin, with each monomer bound to one 1,4‐dioxane molecule (Figure S5). Comparative structural analysis revealed minimal conformational changes upon carbohydrate binding. The Cα root‐mean‐square deviation (RMSD) values between malectin (PDB ID: 9IKP) and its carbohydrate complexes were 0.053 Å (vs. 9IL3), 0.125 Å (vs. 9ILA), and 0.091 Å (vs. 9ILF), indicating that carbohydrate ligands induce negligible structural rearrangement in human malectin (Figure 6H).

### 2.7 Carbohydrate‐Binding Sites of Human Malectin

**FIGURE 7:**
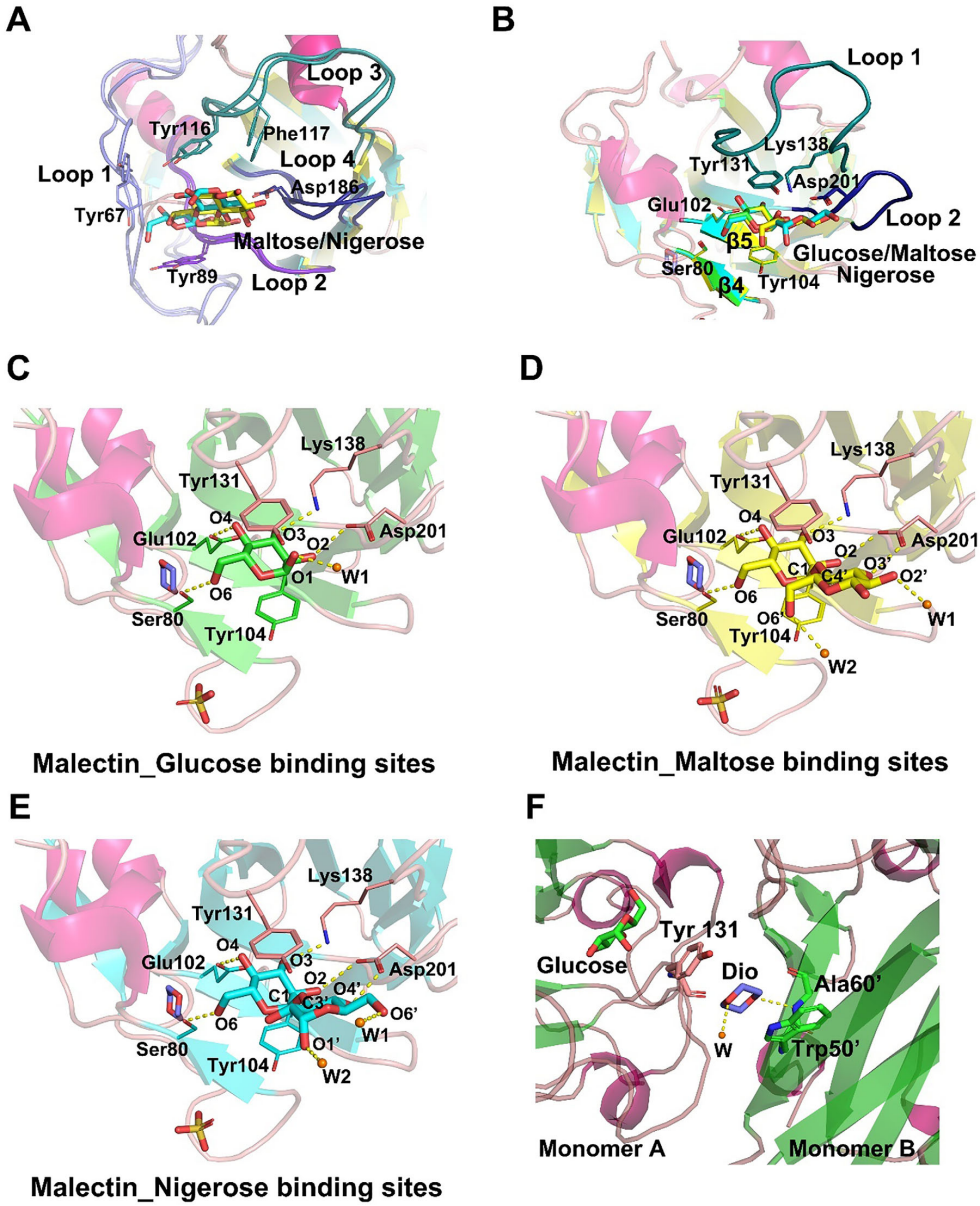
The carbohydrate‐binding sites of human malectin. (A) The carbohydrate‐binding pockets of Xenopus laevis malectin (PDB IDs: 2KR2 for maltose; 2K46 for nigerose). (B) The carbohydrate‐binding pockets of human malectin (PDB IDs: 9IL3 for glucose; 9ILA for maltose; 9ILF for nigerose and 9IKP for no ligand). (C) Carbohydrate‐binding sites in the human malectin‐glucose complex (PDB ID: 9IL3). (D) Carbohydrate‐binding sites in the human malectin‐maltose complex (PDB ID: 9ILA). (E) Carbohydrate‐binding sites in the human malectin‐nigerose complex (PDB ID: 9ILF). (F) The 1,4‐dioxane molecule bound at the interface between monomers A and B in the human malectin‐glucose structure (PDB ID: 9IL3).

The CRD of human malectin exhibits distinct structural features compared to those of Xenopus laevis malectin (Figure 7A,B). Structural analysis reveals that the CRD of Xenopus laevis malectin forms a binding pocket composed of four loops: Loop1 (Gly62–Gly68), Loop2 (Thr86–Asp90), Loop3 (Glu114–Ala118), and Loop4 (Tyr185–Asn187). In contrast, the human malectin CRD features two β‐strands (β4: Arg78–Ser80; β5: Glu102–Tyr104) and two loops (Loop1: Tyr131–Lys138; Loop2: Asp201–Pro203). Notably, two loops in Xenopus laevis malectin are reorganized into β‐strands in the human ortholog, suggesting enhanced conformational stability of the carbohydrate‐binding pocket. The β‐strand‐dominated architecture in human malectin improves ligand stabilization. For instance, glucose, maltose, and nigerose exhibit overlapping binding conformations in the human CRD (Figure 7A,B), whereas these carbohydrates display poor overlap in Xenopus laevis malectin complexes (PDB IDs: 2KR2 for maltose; 2K46 for nigerose). Furthermore, the human CRD retains its β‐strand‐loop architecture even in the unliganded state (Figure 7B), indicating an evolutionary adaptation unique to human malectin.

In the malectin‐glucose complex structure, the side chains of Ser80, Glu102, Lys138, and Asp201 mediate hydrogen bonds with the C6, C4, C3, and C2 hydroxyl groups of glucose, respectively. Additionally, the aromatic rings of Tyr104 and Tyr131 engage in π‐π stacking with the glucose ring (Figure 7C). A water molecule forms a hydrogen bond with the C1 hydroxyl group of glucose, facilitating malectin‐glucose binding. In the malectin‐maltose complex, Ser80, Glu102, Lys138, and Asp201 interact with the C6, C4, C3, and C2 hydroxyl groups of the non‐reducing end glucose moiety via hydrogen bonds. The aromatic rings of Tyr104 and Tyr131 participate in π‐π stacking with the non‐reducing end glucose ring. Notably, Asp201 and two water molecules form hydrogen bonds with the C3, C2, and C6 hydroxyl groups of the reducing end glucose moiety, stabilizing malectin‐maltose interactions (Figure 7D). For the malectin‐nigerose complex, six residues (Ser80, Glu102, Lys138, Asp201, Tyr104, and Tyr131) collectively mediate nigerose binding (Figure 7E). These six residues define the canonical carbohydrate‐binding sites of human malectin, which contrasts with the previously reported binding residues (Tyr67, Glu87, Tyr89, Tyr116, Phe117, and Asp186) in Xenopus laevis malectin (Figure 5C).

Interestingly, a 1,4‐dioxane molecule is observed between monomers A and B in the malectin dimer structure. This molecule, a component of the crystallization solution, was essential for crystal growth, as attempts to crystallize the protein in its absence were unsuccessful. Notably, the 1,4‐dioxane ring engages in π–π stacking with Tyr131 (from monomer A) and Trp50 (from monomer B), and Tyr131 is part of the six‐residue carbohydrate‐binding sites. In addition, Ala60 in monomer B forms hydrogen bonds with the dioxane (Figure 7F). Therefore, we speculate that the binding of 1,4‐dioxane at the dimer interface may influence both carbohydrate‐binding and dimer stability.

### 2.8 Structural Basis of Human Malectin Recognition for Glc2‐N‐Glycan‐Containing Unfolded Glycoproteins

The malectin‐nigerose complex structure elucidates how malectin specifically recognizes Glc2‐N‐glycans on misfolded glycoproteins. During protein folding in the ER, the newly synthesized Glc_3_Man_9_GlcNAc_2_ oligosaccharide undergoes sequential processing. The first step involves α‐glucosidase I‐mediated cleavage of the terminal glucose residue, generating a Glc_2_Man_9_GlcNAc_2_ intermediate with two α1‐3‐linked glucose moieties at the non‐reducing terminus. Nigerose (Glcα1‐3Glc) structurally mirrors the non‐reducing termini of this intermediate. In the malectin‐nigerose complex, Ser80, Glu102, Lys138, and Asp201 form hydrogen bonds with the C6, C4, C3, and C2 hydroxyl groups of the non‐reducing end glucose (Glc‐A), respectively. Tyr104 and Tyr131 engage in π‐π stacking with the glucose ring. Notably, Asp201 and two water molecules mediate hydrogen bonds with the reducing end glucose (Glc‐B) hydroxyls (C3, C2, and C6), stabilizing malectin‐nigerose binding (Figure 7E). The oxygen atom (O1’) of the C1 hydroxyl group of Glc‐B, where the polymannose part of the Glc2‐N‐glycan would be continued. If at the Glc‐B position there was a mannose residue, as in Glc1‐N‐glycan, the stacking interaction would be hindered as there would be an axial hydroxyl group pointing toward Tyr131 and Phe132. Consequently, properly folded glycoproteins with Glc_1_Man_9_GlcNAc_2_ are rapidly trimmed by α‐glucosidase II and recognized by calnexin/calreticulin. In contrast, the misfolded protein retains the Glc_2_Man_9_GlcNAc_2_ structure due to incomplete processing and becomes a recognition target for malectin. Malectin recognizes this intermediate specifically through its carbohydrate‐binding domain, acting as a marker for misfolded proteins. Next, malectin binds to Ribophorin I to form a functional dimer that preserves the misfolded glycoprotein in the endoplasmic reticulum cavity, preventing it from entering the secretion pathway prematurely. Simultaneously, this complex recruits chaperones (e.g., Bip and calnexin) to facilitate refolding or to direct irreversibly misfolded proteins to ER‐associated degradation (ERAD), thereby maintaining proteostasis.

### 2.9 Malectin Overexpression Attenuates HG‐Induced Apoptosis and ER Stress While Enhancing Invasion in HTR‐8/SVneo Cells

**FIGURE 8:**
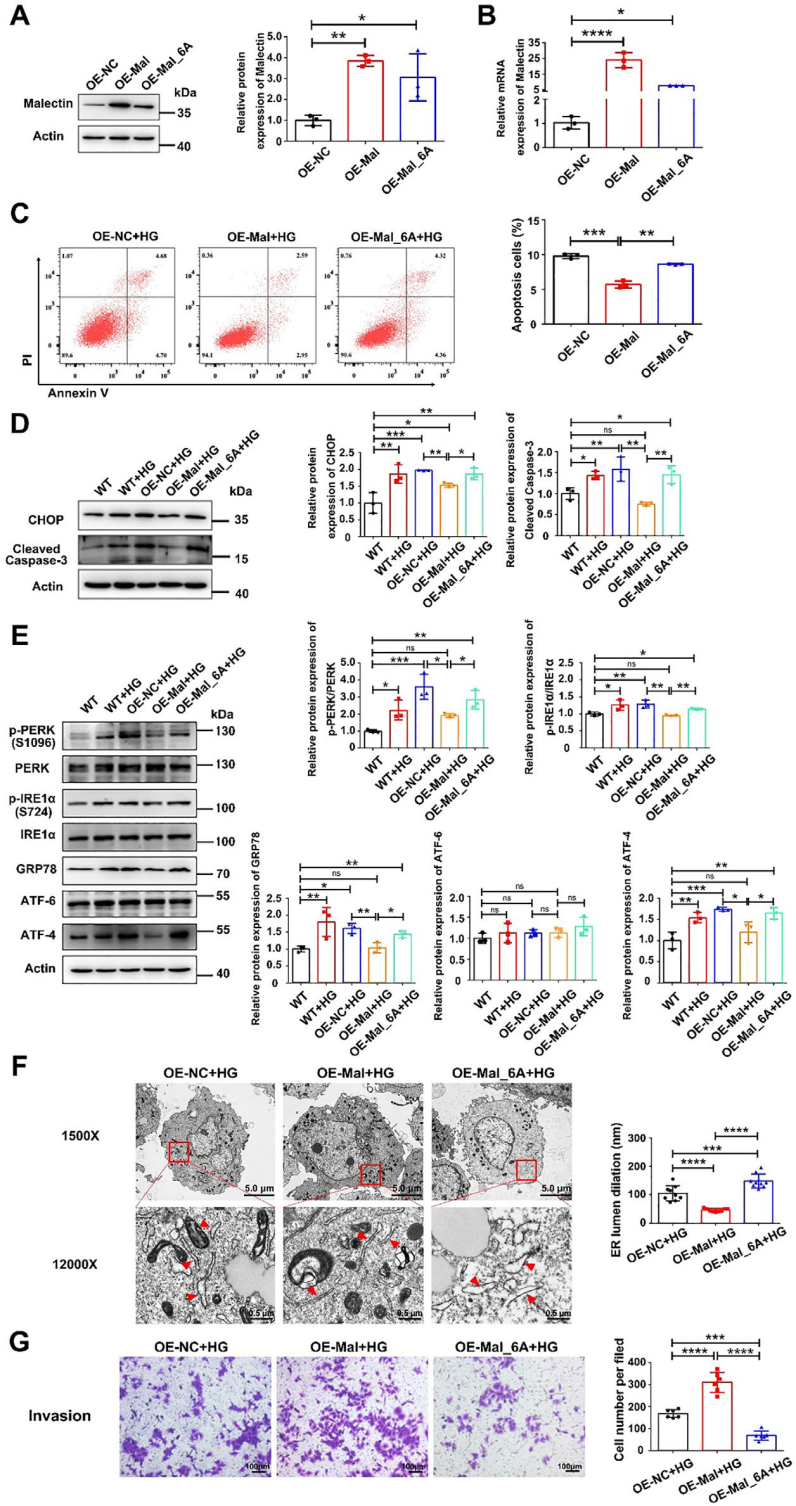
Malectin overexpression attenuates HG‐induced apoptosis and ER stress while enhancing invasion in HTR‐8/SVneo cells. (A,B) Malectin expression levels in OE‐NC, OE‐Mal and OE‐Mal_6A cells were determined by Western blot (band intensities normalized to Actin; n = 3) and qPCR (expression normalized to Actin; n = 3). (C) Apoptosis was assessed by flow cytometry after Annexin V/PI staining (n = 3). The gating strategy involved sequential selection of intact cells based on FSC‐A/SSC‐A, exclusion of doublets using FSC‐H/FSC‐A, and quantification of apoptotic populations (Annexin V+/PI‐ for early apoptosis; Annexin V+/PI+ for late apoptosis/necrosis). (D) Protein levels of CHOP and cleaved caspase‐3 were analyzed by Western blot (normalized to Actin; n = 3). (E) Activation of ER stress pathways was evaluated by Western blot (normalized to Actin; n = 3). (F) ER ultrastructure was examined by TEM; red arrows indicate dilated ER lumen, with quantitative analysis of lumen dilation (n = 9 independent measurements per group). (G) Cell invasion capacity was measured by Transwell assay. Cell counts were conducted in five independent fields of view per group (n = 5). All quantitative data are presented as mean ± SD. Statistical significance was determined by one‐way ANOVA with Tukey's post hoc test (^****^ *p* < 0.0001, ^***^ *p* < 0.001, ^**^ *p* < 0.01, ^*^ *p* < 0.05, ns > 0.05).

We constructed stable HTR‐8/SVneo cell lines overexpressing wild‐type malectin (OE‐Mal) or a mutant malectin (OE‐Mal_6A), in which six carbohydrate‐binding residues identified from the resolved human malectin structure were substituted with alanines. Western blot and qPCR analyses confirmed successful overexpression, with OE‐Mal and OE‐Mal_6A exhibiting approximate increases of approximately 4‐fold and 3‐fold in protein levels, and approximately 25‐fold and 5‐fold in mRNA levels, respectively, relative to the OE‐NC (Figure 8A,B). Immunofluorescence analysis also shows ER localization of overexpressed malectin (Figure S7A,B). The apoptosis of OE‐NC, OE‐Mal, and OE‐Mal_6A cells treated with HG was confirmed by flow cytometry. The apoptosis rate of OE‐NC cells treated with high glucose was 9.76% ± 0.19%, while that of OE‐Mal+HG cells was 5.68% ± 0.29%, approximately half that of OE‐NC+HG cells. The apoptosis rate of OE‐Mal_6A cells treated with high glucose was 8.63% ± 0.03%, a rate similar to that in OE‐NC+HG cells (Figure 8C). Consistent with flow cytometry data, Western blot quantification showed elevated cleaved‐caspase‐3 and CHOP protein levels in HG‐treated OE‐NC cells, which were significantly attenuated in OE‐Mal cells but remained unaltered in OE‐Mal_6A cells (Figure 8D). These findings collectively demonstrate that malectin overexpression protects against HG‐induced apoptosis via a carbohydrate‐binding‐dependent mechanism, whereas the non‐functional mutant fails to mimic this protective effect. As previously shown, malectin knockdown activates the IRE1α and PERK pathways, exacerbating ER stress in HG‐treated HTR‐8/SVneo cells. Here, Western blot analysis revealed that OE‐Mal+HG cells exhibited significantly reduced p‐PERK/PERK and p‐IRE1α/IRE1α ratios compared to OE‐NC+HG cells. In contrast, OE‐Mal_6A+HG cells displayed re‐elevated p‐PERK/PERK and p‐IRE1α/IRE1α ratios, reaching levels comparable to OE‐NC+HG controls. Correspondingly, GRP78 and ATF4 protein levels were downregulated in OE‐Mal+HG cells but upregulated in OE‐Mal_6A+HG cells under HG conditions (Figure 8E). In addition, TEM analysis revealed that Malectin overexpression significantly reduced HG‐induced ER lumen dilation from 105.4 ± 8.79 nm (OE‐NC+HG) to 46.60 ± 1.95 nm (OE‐Mal+HG). In contrast, OE‐Mal_6A+HG exacerbated dilation to 148.2 ± 8.21 nm. Consequently, these results highlight the critical role of Malectin's carbohydrate recognition function in maintaining ER homeostasis (Figure 8F). Furthermore, Malectin overexpression enhanced cell invasion by approximately 2‐fold compared to the OE‐NC group. In contrast, OE‐Mal_6A cells exhibited the weakest invasive capacity, reduced by half relative to the control and representing only one‐fifth of the OE‐Mal group's level under HG conditions (Figure 8G). These results collectively establish that Malectin overexpression attenuates HG‐induced apoptosis and ER stress while enhancing invasion in HTR‐8/SVneo cells, and that its six conserved carbohydrate‐binding residues are essential for mediating these functions.

### 2.10 Malectin Overexpression Attenuates HG‐Induced ER Stress While Enhancing Syncytialization and Glucose Uptake in BeWo Cells

**FIGURE 9:**
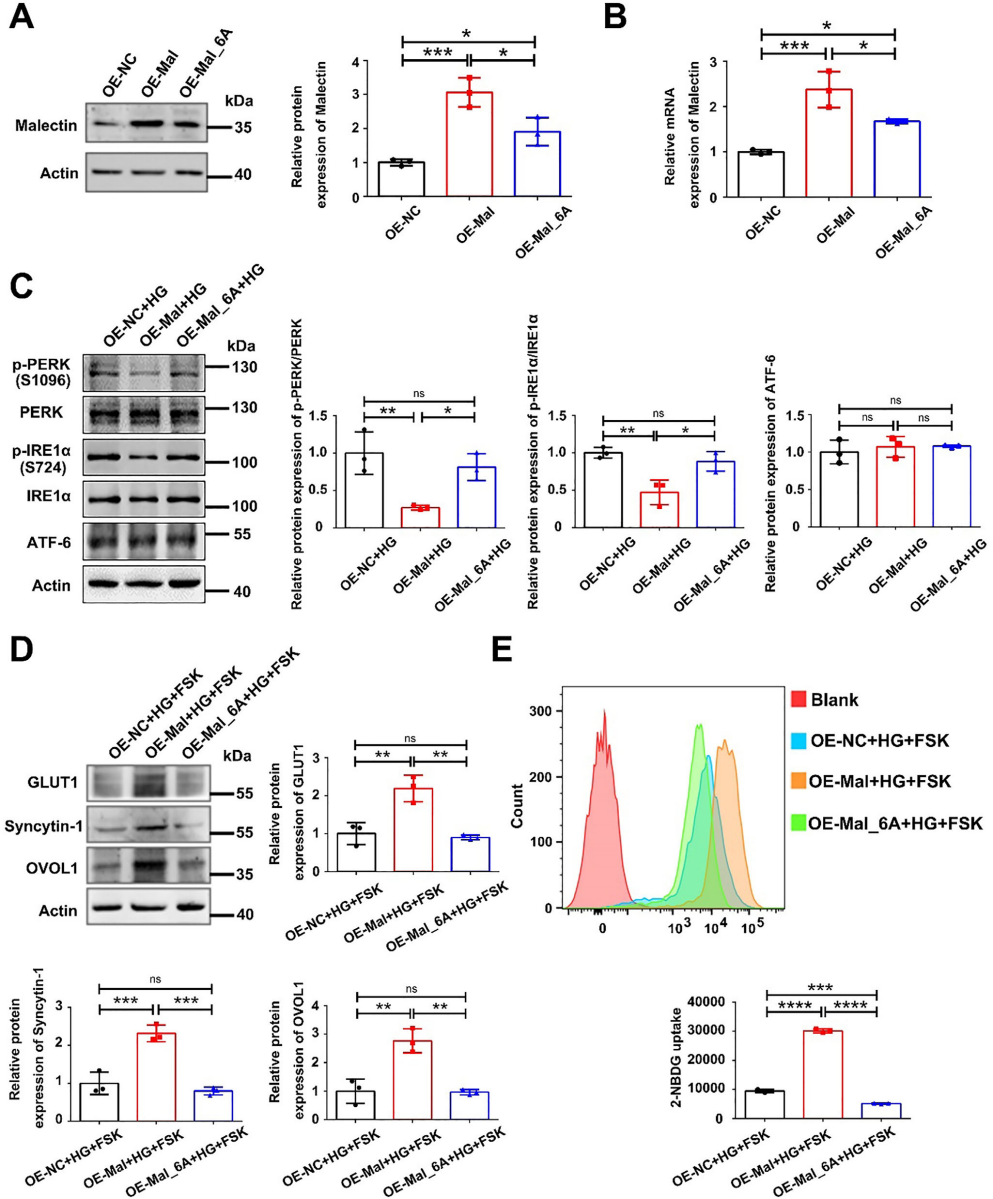
Malectin overexpression attenuates HG‐induced ER stress while enhancing syncytialization and glucose uptake in BeWo cells. (A,B) Overexpression efficiency was validated by Western blot with band intensities normalized to Actin and qPCR with expression normalized to Actin. (C) Activation of ER stress pathways was evaluated by Western blot (normalized to Actin). (D) Western blot analysis of syncytialization and GLUT1 expression levels (normalized to Actin). (E) Glucose uptake was measured by flow cytometry using 2‐NBDG. All quantitative data are presented as mean ± SD from n = 3 biologically independent experiments. Statistical significance was determined by one‐way ANOVA with Tukey's post hoc test (^****^ *p* < 0.0001, ^***^ *p* < 0.001, ^**^ *p* < 0.01, ^*^ *p* < 0.05, ns > 0.05).

The previous results show that malectin knockdown exacerbates ER stress and attenuates syncytialization and glucose uptake in HG‐treated BeWo cells. Here, we also constructed stable BeWo cell lines overexpressing malectin or its mutant (OE‐Mal and OE‐Mal_6A). Western blot and qPCR analyses verified successful overexpression, with OE‐Mal and OE‐Mal_6A showing increases of approximately 3‐ and 2‐fold at the protein level, and approximately 2.5‐ and 2‐fold at the mRNA level, respectively, compared to OE‐NC (Figure 9A,B). Immunofluorescence analysis also shows ER localization of overexpressed malectin (Figure S7C,D). Malectin knockdown activates the IRE1α and PERK pathways, exacerbating ER stress in HG‐treated BeWo cells. Western blot analysis revealed that compared to the OE‐NC+HG cells, the p‐PERK/PERK and p‐IRE1α/IRE1α ratios were significantly reduced in OE‐Mal+HG cells, whereas the OE‐Mal_6A+HG cells showed further increased ratios, reaching levels comparable to the control (Figure 9C). These results demonstrate that Malectin overexpression alleviates HG‐induced ER stress in BeWo cells, consistent with its protective role observed in HTR‐8/SVneo cells. Malectin knockdown attenuates syncytialization and glucose uptake in HG‐treated BeWo cells, whereas malectin overexpression effectively reverses this impairment, demonstrating its protective role. Specifically, the OE‐Mal cells showed elevated expression of Syn‐1, OVOL‐1, and GLUT1 (approximately 2‐ to 3‐fold higher than OE‐NC cells), while the OE‐Mal_6A cells failed to enhance expression under HG+FSK conditions (Figure 9D). Consistent with these findings, flow cytometry revealed an approximately 3‐fold increase in glucose uptake in OE‐Mal cells, whereas OE‐Mal_6A cells exhibited no promotive effect (Figure 9E). These results indicate that malectin promotes BeWo cell fusion and glucose uptake under HG conditions via its six carbohydrate‐binding sites.

### 2.11 Glycoprotein Quality Control by Malectin is Mediated by Six Key Carbohydrate‐Binding Residues

**FIGURE 10:**
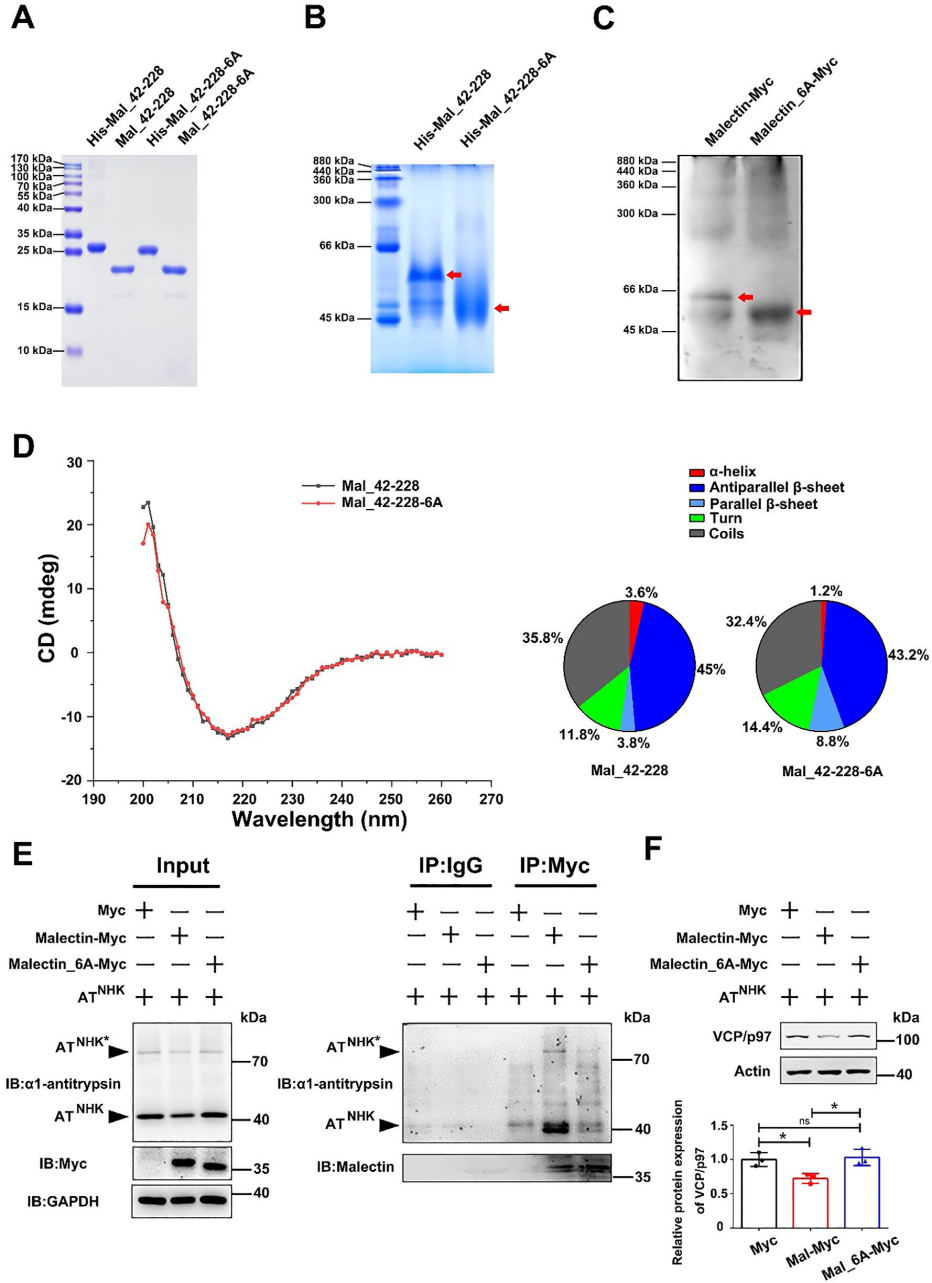
Glycoprotein quality control by malectin is mediated by six key carbohydrate‐binding residues. (A) SDS‐PAGE analysis of malectin and malectin_6A. (B) Blue‐native‐PAGE analysis of His‐Mal_42‐228 and His_42‐228_6A. The red arrow indicates the position of the corresponding dimer band. (C) Blue‐native‐PAGE analysis of Malectin‐Myc and Malectin_6A‐Myc in HTR‐8/Svneo cells. The red arrow indicates the position of the corresponding dimer band. (D) The CD spectrum and proportion of secondary structure content of malectin and malectin_6A. (E) Co‐IP analysis of the binding activity of malectin and malectin_6A to AT^NHK^. AT^NHK*^ represents the dimeric form of AT^NHK^. (F) Western blot analysis of VCP expression levels (normalized to Actin, n = 3). All quantitative data are presented as mean ± SD. Tatistical significance was determined by one‐way ANOVA with Tukey's post hoc test for multiple comparisons (^*^ *p* < 0.05, ns > 0.05).

Functional analyses demonstrated that Malectin mediated protective effects against HG‐induced damage in both HTR‐8/SVneo and BeWo cells, whereas the Malectin_6A mutant lacked this protective function. To confirm that this functional loss was not due to mutation‐induced structural misfolding, we expressed and purified Malectin_6A in vitro (Figure 10A). Circular dichroism (CD) analysis revealed nearly identical spectral profiles between Malectin and Malectin_6A, indicating that the six residues mutation did not alter the overall folding or secondary structure of Malectin (Figure 10D). To further corroborate the structural integrity at the tertiary and quaternary levels, we performed computational modeling. The three‐dimensional structure of the Malectin_6A dimer was predicted using SWISS‐MODEL with the wild‐type malectin crystal structure (PDB: 9IKP) as a template. The high‐quality model (GMQE score = 0.8) confirmed that Malectin_6A also exists as a homodimer. Each monomer retains the characteristic 14 β‐strands and 5 α‐helices. Superposition of the Malectin_6A model onto the wild‐type structure yielded a backbone Cα RMSD of 0.057 Å, demonstrating that the mutations did not perturb the global protein architecture (Figure S6A,B). Analysis of the dimer interface revealed that it is stabilized by 11 hydrogen bonds in both the wild‐type and the mutant. In the wild‐type, these involve six residues from monomer A (Ile49, His66, Arg68, Lys69, Pro114, Lys115) and eight from monomer B (Tyr82, Tyr131, Ala133, Gln134, Ser135, Gln136, Gln137, His161). Notably, one of the mutated residues, Tyr131, participates in this network only via hydrogen bonds from its backbone carbonyl oxygen to Arg68, while its side chain is not involved. In the Malectin_6A model, the interface is identically formed, with Tyr131 simply replaced by Ala131. Therefore, the six mutations do not interfere with the inter‐subunit interactions required for dimer stability (Figure S6C–E).

To elucidate the native oligomeric state of malectin, we performed Blue‐Native PAGE analysis. Purified recombinant His‐Malectin_42‐228 displayed a prominent band at approximately 60 kDa under non‐denaturing conditions, consistent with its expected dimeric molecular weight (monomer ∼27 kDa). Intriguingly, His‐Malectin_42‐228_6A, migrated slightly faster, forming a major band at approximately 50 kDa. Notably, a faint band at 50 kDa was also detectable for the wild‐type protein (Figure 10B). A highly congruent pattern was observed in eukaryotic cells. Overexpressed Malectin‐Myc in HTR‐8/SVneo cells showed a distinct band at approximately 66 kDa, corresponding to a dimer (monomer ∼33 kDa), while the Malectin_6A‐Myc mutant band migrated to a slightly lower position (Figure 10C). Collectively, these in vitro and cellular data indicate that malectin exists as a dimer irrespective of the expression system, but the 6A mutation alters its migration behavior in BN‐PAGE. Prior CD spectroscopy confirmed that the 6A mutation does not disrupt the core secondary structure, indicating proper protein folding. The computational model further establishes that the tertiary and quaternary structures are preserved. Furthermore, this mutation does not affect its sole predicted N‐glycosylation site at Asn‐268. Therefore, the observed difference in electrophoretic mobility is not attributable to protein misfolding or altered glycosylation. A plausible explanation is that the 6A mutation modifies the surface charge or hydrophobicity of the protein, consequently affecting its interaction with the G‐250 dye integral to BN‐PAGE, leading to an apparent molecular weight shift. This is supported by the calculated electrostatic surface potential, which shows a localized change in charge distribution, with the potential range shifting from −63.66 to +63.66 kT/e for the wild‐type to −59.44 to +59.44 kT/e for the mutant (Figure S6F,G). The faint 50 kDa band in the wild‐type sample may represent a minor population of dimers in a surface state analogous to that of the mutant. Chemical cross‐linking experiments further confirmed that both wild‐type and mutant Malectin_6A can form dimers (Figure S5C), these finding consistent with our previously solved malectin crystal structure presenting as a dimer. These results confirm that malectin exists as a dimer and the specific disruption of carbohydrate‐recognition and binding activity of malectin_6A. Co‐IP assay further demonstrated that, unlike wild‐type Malectin which efficiently bound misfolded glycoprotein AT^NHK^ (α1AT variant) and reduced levels of the ERAD activation marker VCP, the Malectin_6A mutant lost the ability to interact with AT^NHK^ and consequently failed to suppress the compensatory activation of the ERAD pathway (Figure 10E,F). These results collectively indicate that the six key residues constitute the functional core of Malectin's carbohydrate recognition domain (CRD), and that carbohydrate‐mediated glycoprotein binding is the molecular basis for its quality control function.

### 2.12 Therapeutic Effect of Malectin on Hyperglycemia and Placental ER Stress in GDM Mice

**FIGURE 11:**
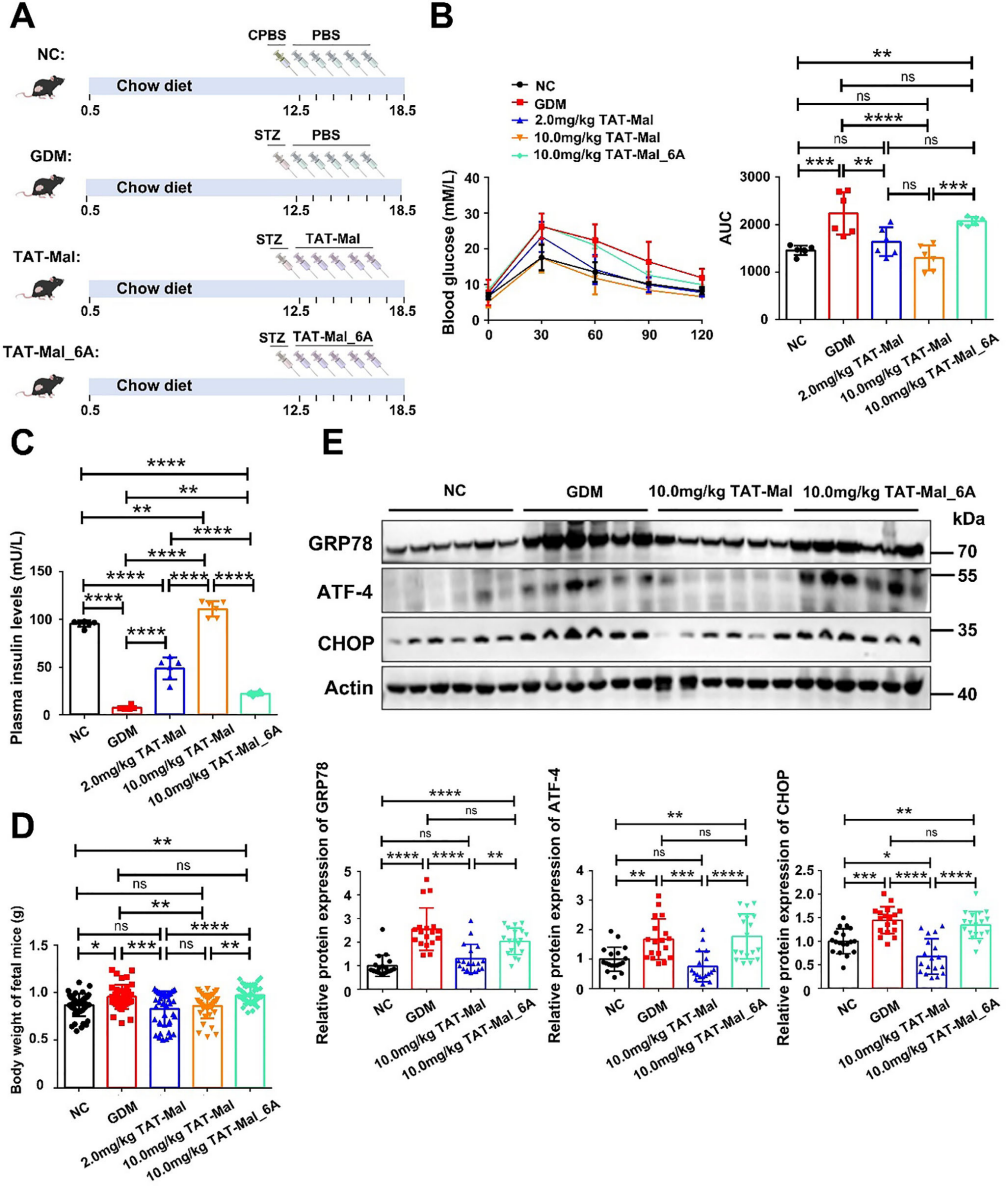
Therapeutic alleviation of GDM mice hyperglycemia and placental ER stress by malectin. (A) Schematic illustration of the animal experimental design. NC stands for WT mice only treated with sodium citrate buffer (CPBS), GDM stands for WT mice treated with STZ. TAT‐Mal stands for GDM mice treated with TAT‐Mal, and TAT‐Mal_6A stands for GDM mice treated with TAT‐Mal_6A. (B) OGTT curves of pregnant mice in NC, GDM, 2.0 mg/kg TAT‐Mal, 10.0 mg/kg TAT‐Mal, and 10.0 mg/kg TAT‐Mal_6A groups and the area under curve (AUC), n = 6 mice per group. (C) The content of insulin was analyzed by ELISA, n = 6 mice per group. (D) Fetal weight in NC (n = 44), GDM (n = 38), 2.0 mg/kg TAT‐Mal (n = 39), 10.0 mg/kg TAT‐Mal (n = 38), and 10.0 mg/kg TAT‐Mal_6A (n = 47). (E) Protein expression levels of the ER stress markers GRP78, ATF4, and CHOP in placental tissues from NC, GDM, 10.0 mg/kg TAT‐Mal, and 0.0 mg/kg TAT‐Mal_6A were determined by Western blot. For Western blot quantification, band intensities were normalized to Actin. n = 18 placenta per group. Statistical significance was determined by one‐way ANOVA with Tukey's post hoc test. All data are presented as the means ± SD. (^****^ *p* < 0.0001, ^***^ *p* < 0.001, ^**^ *p* < 0.01, ^*^ *p* < 0.05, ns > 0.05).

Building on the protective effects of malectin observed in GDM cell models, we further evaluated its therapeutic potential in vivo using an STZ‐induced GDM mouse model. To enable efficient cellular delivery and ER targeting, we engineered a TAT‐2HA‐malectin‐KDEL fusion protein (TAT‐Mal), in parallel, a TAT‐Mal_6A group, administered with the mutant TAT‐Mal_6A protein, was included as a control (Figure S8B,E). Preliminary validation confirmed that TAT‐Mal and TAT‐Mal_6A effectively entered HTR‐8/SVneo cells and localized to the ER (Figure S8C,D,F,G). GDM mice were intraperitoneally administered TAT‐Mal or TAT‐Mal_6A to assess their protective efficacy, with the experimental timeline and treatment scheme outlined in Figure 11A. Oral glucose tolerance tests (OGTTs) were performed on five groups of mice at E17.5. As shown in Figure 11B, GDM mice exhibited significantly higher blood glucose levels at all time points (0–120 min) compared to normal controls. Treatment with TAT‐Mal significantly attenuated hyperglycemia in a dose‐dependent manner. At 30 min, the 10.0 mg/kg TAT‐Mal group already showed glucose levels comparable to normal controls (*p* > 0.9999 vs. control; *p* < 0.0001 vs. GDM), and the 2.0 mg/kg group achieved similar normalization by 60 min (*p* = 0.9781 vs. control; *p* < 0.0001 vs. GDM). In contrast, blood glucose levels in the TAT‐Mal_6A‐treated group remained comparable to the GDM group at both 30 and 60 min (*p* = 0.9998 and *p* = 0.806, respectively). Consistent with these observations, the area under the blood glucose curve (AUC) was markedly elevated in GDM mice (*p* = 0.0004) but was significantly reduced by TAT‐Mal treatment at both 2.0 mg/kg (*p* = 0.0069) and 10.0 mg/kg (*p* < 0.0001) doses, returning to levels comparable to the normal group (*p* = 0.738 and *p* = 0.8054, respectively). However, the AUC in the TAT‐Mal_6A group showed no significant difference from the GDM group (*p* = 0.8305), but it remained significantly higher than that in the 10.0 mg/kg TAT‐Mal group (*p* = 0.0005). These results indicate that TAT‐Mal effectively ameliorates hyperglycemia in GDM mice, with a faster onset of action at the higher dose. On gestational day 18.5, mice from all five groups were euthanized for analysis of insulin content, offspring weight, and placental ER stress, and provided critical evidence for the efficacy of malectin. As expected, STZ treatment severely impaired pancreatic β‐cell function, resulting in hypoinsulinemia (7.3 ± 1.9 mu/L) in the GDM group. Administration of TAT‐Mal at 2.0 mg/kg significantly restored insulin levels (48.4 ± 11.59 mU/L). Strikingly, the high‐dose group (10.0 mg/kg) exhibited insulin levels comparable to, or even slightly exceeding, those of the normal controls (110.9 ± 8.14 mU/L vs. 95.43 ± 3.4 mU/L). The above‐normal insulin level observed in the high‐dose group may reflect a compensatory hyperinsulinemia resulting from significantly improved β‐cell survival and function. In contrast, administration of the high‐dose (10 mg/kg) mutant TAT‐Mal_6A failed to restore insulin secretion, yielding only a marginal increase (22.22 ± 2.19 mU/L) over the GDM group, a level substantially lower than that achieved by wild‐type TAT‐Mal (Figure 11C). Offspring of GDM mice exhibited significantly higher body weight (0.96 ± 0.13 g) than those from the normal pregnancy group (0.87 ± 0.12 g). In contrast, offspring from both the 2.0 mg/kg (0.83 ± 0.18 g) and 10.0 mg/kg (0.86 ± 0.13 g) TAT‐Mal treatment groups showed significantly reduced weights, comparable to the normal group, indicating that malectin effectively protects against aberrant fetal growth in GDM. However, administration of the high‐dose (10 mg/kg) mutant TAT‐Mal_6A failed to correct this fetal overgrowth, as the body weight of offspring in this group (0.97 ± 0.09 g) remained comparable to that of the untreated GDM group (Figure 11D). Assessment of placental ER stress revealed significantly elevated levels of the markers GRP78, ATF‐4, and CHOP in GDM mice—approximately twice those observed in the normal group. In contrast, placentas from the 10 mg/kg TAT‐Mal‐treated group exhibited markedly reduced expression of these proteins, returning to levels comparable to the normal group. However, administration of the high‐dose (10 mg/kg) mutant TAT‐Mal_6A failed to attenuate placental ER stress, as the expression levels of GRP78, ATF‐4, and CHOP remained comparable to those in the untreated GDM group (Figure 11E). In addition, TAT‐Mal was detected in multiple organs—including the liver, spleen, lungs, kidneys, and placenta—of treated mice, with higher accumulation observed in the spleen, kidneys, and placenta than in the liver and lungs (Figure S8H). We propose that by robustly mitigating high glucose‐induced ER stress via its glycoprotein quality control function, malectin not only prevents β‐cell apoptosis but also creates a microenvironment conducive to functional recovery. Overall, these results demonstrate that malectin effectively alleviates hyperglycemia and placental ER stress in GDM mice, highlighting its promising therapeutic potential. Importantly, these beneficial effects are highly dependent on its intact carbohydrate recognition and binding capability.

## 3 Discussion

​The pathogenesis of GDM remains incompletely understood, necessitating elucidation of novel pathological mechanisms and innovative therapeutic strategies. Mounting evidence indicates that ER stress not only drives oncogenesis and neurodegenerative diseases but also constitutes a key pathological mechanism in metabolic disorders, including diabetes mellitus and obesity [38, 39]. The placenta, a metabolically active organ during pregnancy, contains trophoblast cells with exceptionally high endoplasmic reticulum content due to their intense protein synthesis demands. ER stress‐induced dysfunction of trophoblasts has been implicated in pregnancy complications such as preeclampsia, fetal growth restriction, and GDM [15]. Notably, ER stress is activated during the early stages of insulin resistance development and exhibits a bidirectional regulatory relationship with obesity and type 2 diabetes mellitus [40]. Consequently, targeting ER stress attenuation to mitigate hyperglycemia‐induced trophoblast damage may represent a novel therapeutic avenue for GDM. Malectin, an endoplasmic reticulum‐resident lectin, plays a pivotal role in glycoprotein quality control by binding to the Glc2‐N‐glycan structure retained on misfolded proteins. However, whether malectin alleviates ER stress pathogenesis and GDM progression through its glycoprotein quality control function remains to be fully established.

Our findings revealed significantly elevated protein and mRNA levels of malectin in placental tissues from GDM patients compared to normal controls. Consistent with these observations, malectin expression was markedly upregulated in HG‐treated HTR‐8/SVneo cells, suggesting its aberrant activation and may be involved in the pathogenesis of GDM. Malectin knockdown significantly reduced the invasive ability and exacerbated apoptosis of HTR‐8/SVneo cells in HG condition. Transcriptomic sequencing further demonstrated that malectin deficiency induced robust upregulation of UPR‐related genes, ER stress genes and ER stress‐induced apoptotic genes. Furthermore, knockdown of malectin significantly reduced the syncytialization and glucose uptake capacity of BeWo cells in HG condition. Notably, HG‐ induced ER stress in both HTR‐8/SVneo and BeWo cells, accompanied by activation of the PERK and IRE1α signaling pathways. In HTR‐8/SVneo cells, the ATF‐6 pathway remained unaltered, whereas in BeWo cells, all three UPR branches were activated. Knockdown of malectin exacerbated HG‐induced ER stress and further enhanced the activation of PERK and IRE1α in both cell lines, while the ATF‐6 pathway remained unaffected. A review of the literature further reveals that the three branches of the UPR can be activated in a differential manner. For instance, HG treatment in HUVEC cells selectively activates the PERK and IRE1α pathways, inducing ER stress without reported involvement of ATF‐6 [41]. Similarly, cytokines such as IL‐1β and IFN‐γ activate ER stress via PERK and IRE1α, but not ATF‐6, consistent with our observations [42]. In contrast, He et al. reported that Protein arginine methyltranferase‐1 (PRMT‐1) knockdown reduced HG‐induced activation of both PERK and ATF‐6 in HK2 cells, while IRE1α remained unaffected [43]. Collectively, these reports, together with our data​​ showing distinct UPR activation patterns in HTR‐8/SVneo and BeWo cells under high glucose, these findings suggest that the activation profile of the UPR is highly dependent on cell type and the specific nature of the stress stimulus.

To elucidate the molecular mechanism by which malectin binds Glc2‐N‐glycan to modulate ER stress, we resolved the crystal structures of human malectin in its unliganded state (PDB: 9IKP) and in complexes with glucose (PDB: 9IL3), maltose (PDB: 9ILA), and nigerose (PDB: 9ILF). Our structural and biochemical data demonstrate that malectin specifically recognizes glucose, maltose, and nigerose, but not monosaccharides such as mannose or galactose. This raises a key question: What is the physiological relevance of this binding specificity in the placental context of GDM? Notably, the structure of nigerose (Glcα1‐3Glc) structurally mirrors the non‐reducing terminal disaccharide motif (Glcα1‐3Glc) of the Glc_2_Man_9_GlcNAc_2_ glycan intermediate in the ER. This suggests that malectin's binding to nigerose likely reflects its innate capacity to recognize aberrant glycan structures during glycoprotein quality control, thereby linking our in vitro observations to its physiological role in mitigating ER stress in GDM placentas. The specificity of malectin for glucosyl‐based motifs—while excluding other common monosaccharides—ensures precise targeting of glycoprotein folding intermediates without interfering with unrelated glycosylation pathways, underscoring its functional selectivity. We acknowledge that native ligands in vivo are likely more complex than simple disaccharides and may involve specific glycan structures on fully folded glycoproteins. Nevertheless, the binding properties uncovered here provide crucial mechanistic insight into malectin's recognition mechanism, offering a foundation for future studies exploring its interactions with diverse glycoprotein substrates in metabolic and gestational diseases. Furthermore, structural analysis identified six critical carbohydrate recognition and binding sites (Ser80, Glu102, Tyr104, Tyr131, Lys138, Asp201) in human malectin, which differ from those reported in Xenopus laevis malectin. Based on these structural insights, we designed malectin_6A (Mal_6A) mutant in which six carbohydrate‐binding sites were mutated to alanine. Functional assays demonstrated that wild‐type malectin overexpression (OE‐Mal) significantly alleviated cell damage induced by high glucose in HTR‐8/SVneo and BeWo cells, whereas OE‐Mal_6A abolished these protective effects, confirming the functional necessity of the CRD in human malectin. We further evaluated its therapeutic potential in vivo using an STZ‐induced GDM mouse model. Administration of TAT‐Malectin demonstrates therapeutic efficacy in GDM mice by increasing insulin secretion, reducing hyperglycemia, and preventing macrosomia in offspring. This identifies malectin as a key placental protector and a promising protein therapeutic candidate for GDM. In summary, our results indicate that malectin upregulation in GDM placentas represents an adaptive compensatory mechanism to mitigate high glucose‐induced damage. Mechanistically, malectin recognizes Glc2‐N‐glycans on misfolded glycoproteins via its CRD, thereby enhancing glycoprotein quality control, attenuating ER stress and damage, and preserving trophoblast function. The efficacy of exogenous malectin supplementation in ameliorating GDM phenotypes in vivo further highlights its potential as a therapeutic target.

Interestingly, structural analysis revealed a 1,4‐dioxane molecule positioned at the dimer interface of human malectin, a component essential for successful crystallization. The molecule engages in specific interactions with key residues—including π‐π stacking with Tyr131 (monomer A) and Trp50 (monomer B), and hydrogen bonding with Ala60 (monomer B)—several of which are part of the conserved carbohydrate‐binding site. These observations suggest that the binding of 1,4‐dioxane could potentially​ modulate malectin's carbohydrate‐binding and dimerization functions. Given its strategic binding location and interactions, ​​the 1,4‐dioxane molecule​​ represents a promising scaffold for developing malectin‐targeted agonists. Although 1,4‐dioxane itself exhibits neurotoxicity and carcinogenicity risks, its chemical modifiability enables the design of safer analogs [44]. Notably, structural optimization of 1,4‐dioxane derivatives has demonstrated significant therapeutic potential in targeting the 5‐hydroxytryptamine (5‐HT) receptor, particularly in the field of mental illness treatment and antitumor activity in prostate cancer [45, 46]. Furthermore, Fabio Del Bello et al. reported that 1,4‐dioxane as a suitable scaffold for the development of novel M3 muscarinic receptor antagonists [47]. Therefore, 1,4‐dioxane derivatives with low toxicity and high affinity targeting malectin may have therapeutic potential for GDM.

Plant malectin‐like receptor kinases (MLRs), also designated as Catharanthus roseus receptor‐like kinase‐1‐like proteins (CrRLK1Ls), ​​have been well‐characterized in pollen tube reception, tip growth regulation, cell wall integrity sensing, hormonal signaling, and plant immunity [48, 49]. In contrast, animal malectins predominantly function in glycoprotein quality control within the ER. Notably, plant and animal malectins exhibit significant divergence in protein sequences, structural architectures, and carbohydrate recognition domains. Unlike animal malectins, plant MLRs lack residues critical for diglucoside binding, suggesting distinct ligand‐binding mechanisms [50, 51]. It is speculated that animals and plants have undergone adaptive evolution of malectin in the face of different environmental pressures. Sequence conservation analysis reveals that animal malectins are highly conserved across vertebrate species. For instance, human malectin shares 83.7%, 95.16%, 95.85%, and 75.63% sequence identity with its orthologs in Xenopus laevis, mouse, rat, and zebrafish, respectively (Figure 5C). The high sequence conservation underscores the functional importance and evolutionary constraint of malectin in maintaining core biological processes.

Glycosylation is ​​one of the most diverse​​ protein modification by which complex glycan structures are added to protein or lipid backbones [52]. Contrary to glycation, which has been usually connected to diabetes [53], glycosylation is a multi‐stage enzymatic process regulated by an extensive network of genes, resulting in branched complex structures made of monosaccharide units linked by chemical bonds. Proper glycosylation is important for correct protein folding, cell structure maintenance, receptor–ligand interactions, cell signalling, cell–cell recognition and immune defence. Glycosylation changes are well known to influence protein functions [52]. N‐glycosylation changes have been described in different diseases, including type 1 diabetes [54], type 2 diabetes [24], and gestational diabetes [27]. Lee et al. reported that glycosylation failure of glycodelin‐A in Gestational Diabetes Mellitus, which is an abundant decidual glycoprotein with glycosylation‐dependent immunomodulatory activities [27]. In recent years, studies of gestational diabetes using mass spectrometry‐based proteomic technologies showed several glycosylation failure proteins are involved in the regulation of insulin or indirect signaling pathways [28]. Whether malectin participates in the quality control of glycodelin‐A or other glycoproteins within insulin signaling cascades remains to be elucidated.

Limitations and future perspectives of this study. First, our in vitro GDM model was established using high glucose treatment of trophoblast cells. While this model is widely employed in GDM‐related research [55, 56, 57], the clinical pathology of GDM involves a more complex interplay of factors, including hyperglycemia, insulin resistance, and inflammatory cytokines. Future studies utilizing more comprehensive models that integrate these multifaceted factors will provide deeper insights into the disease mechanisms. Second, the 25 mm glucose concentration used in our in vitro experiments exceeds the typical pathological range observed in GDM. Although our glucose gradient experiments demonstrated a clear dose‐dependent effect: PI3K/AKT/GLUT4 signaling was progressively inhibited, apoptosis was gradually increased, and malectin expression was correspondingly upregulated with increasing glucose concentration. A key finding was that the effects of 20 and 25 mm glucose were similar across all experiments, with the exception of a difference in PI3K expression. In addition, although​​ a considerable number of studies have used 25 mm glucose [55, 56, 57], we acknowledge that 20 mm represents a concentration more closely approximates​​ the in vivo pathological range. Therefore, future investigations will prioritize using 20 mm glucose to enhance the physiological relevance of our cellular models. Third, we acknowledge that a larger sample size would further enhance statistical power; however, due to ethical and practical constraints inherent in clinical placental research, obtaining a large cohort is challenging. Importantly, the conclusions derived from our clinical samples (n = 10 per group) are strongly corroborated by subsequent in vitro experiments in two placental cell lines and in vivo studies in a GDM mouse model, ​collectively providing​​ robust mechanistic validation. It is important to note that this study is based on the well‐established, high specificity of malectin for its canonical ER ligand, the G2M9 oligosaccharide. We must clearly state that the present work did not investigate the existence of alternative interacting partners for malectin in non‐physiological, complex in vitro settings; exploring any potential functions beyond its classic glycoprotein quality control pathway represents a distinct and separate avenue for future research. In addition, non‐enzymatic “glycation” reactions (e.g., formation of HbA1c), resulting from chronic hyperglycemia, are an important pathological feature in diabetes [53]. It should be clarified that malectin, as an ER‑resident lectin, functions by specifically recognizing the G2M9 N‑glycan structure on nascent glycoproteins and participating in their folding quality control. This is distinct in both chemical nature and biological significance from the non‑specific modification of protein amino groups by glucose in “glycation” reactions. Nevertheless, ERS is a key common pathway contributing to pancreatic β‑cell dysfunction and apoptosis in various forms of diabetes, including type 1 diabetes (T1DM) and type 2 diabetes (T2DM) [58]. In T2DM, glucolipotoxicity can induce persistent and unresolvable ERS, ultimately leading to β‑cell failure [59]. ERS also plays a significant role in autoimmune‑mediated β‑cell destruction in T1DM [60]. Therefore, we speculate that the mechanism mediated by malectin—alleviating ERS by promoting refolding or targeting misfolded glycoproteins for degradation—may not be limited to GDM but could have broader implications in the pathophysiology of diabetes. Future studies are warranted to further examine the expression, function, and impact of malectin on β‑cell survival and insulin secretion in cellular or animal models of T1DM and T2DM. Additionally, investigating whether prolonged hyperglycemic conditions modulate malectin function or expression through effects on ER homeostasis or protein glycosylation processes would be an interesting direction for future research.

This study provides the first evidence that malectin protects placental trophoblasts from high glucose‐induced ER stress and damage through glycoprotein quality control mediated by its six key carbohydrate‐binding residues. Importantly, intraperitoneal administration of TAT‐Malectin improved glucose metabolism in a GDM mouse model, demonstrating therapeutic potential. These findings establish malectin as both a key endogenous placental protector and a promising protein therapeutic candidate, offering a novel target and therapeutic strategy for GDM.

## 4 Experimental Section

### 4.1 Tissue Collection and Preparation

Human placental tissues were collected from pregnancies between 36 and 42 weeks of gestation at the Xuzhou Maternity and Child Health Care Hospital (Xuzhou, China). The study included two cohorts: a normoglycemic control group (n = 10) and a GDM group (n = 10). Participants with pre‐existing medical comorbidities, including pregestational diabetes, polycystic ovary syndrome (PCOS), preeclampsia (PE), or macrovascular complications, were excluded. This study was approved by the Ethics Committee of Xuzhou Maternity and Child Health Care Hospital ([2023] Lunshen No. 24), and all patients provided written informed consent for this study. GDM diagnosis adhered to the criteria established by the International Association of Diabetes and Pregnancy Study Groups (IADPSG). All pregnancies underwent standardized GDM screening, with the normoglycemic control group defined by negative screening results.

### 4.2 Cell Culture and Treatments

The HTR‐8/SVneo trophoblast cell line (obtained from MeisenCTCC, Zhejiang, China) was cultured in RPMI‐1640 medium (BioChannel Biotechnology Co., Ltd, China). The human choriocarcinoma cell line BeWo was purchased from Haixing Biosciences (China) and cultured in Ham's F12K medium (KeyGEN BioTECH, China). ​​Both media were supplemented​​ with 10% (v/v) fetal bovine serum (TianHang, China) and 1% penicillin‐streptomycin solution (Millipore Sigma) at 37°C in a 5% CO_2_ humidified incubator. To simulate the hyperglycemic environment characteristic of GDM, cells were treated with 25 mm glucose (final concentration) for 48 h in vitro as a specific model of GDM. Normoglycemic controls were maintained in an identical medium containing 5.5 mm glucose. To induce syncytialization, BeWo cells were treated​​ with 20 µm forskolin (FSK) for 24 h.

### 4.3 Plasmid Construction, Lentivirus Packaging, and Infection

pLVshRNA‐EGFP(2A)‐Puro vector was used to suppress the endogenous MLEC gene (Gene ID:9761). The following short hairpin RNA (shRNA) sequences were used: a non‐targeting control (Sh‐NC: 5’‐CCTAAGGTTAAGTCGCCCTCGCTCGAGCGAGGGCGACTTAACCTTAGGTTTTT ‐3’) and a sequence​ targeting MLEC (Sh‐MLEC‐1: 5’‐ GGATATCTTTGATCGTGTTGG ‐3’). To assess potential off‐target effects, a second independent shRNA targeting MLEC (5’‐ GGTACTATGACAATCCCAAGG ‐3’) was also designed and utilized. pCDH‐CMV‐MCS‐EF1‐GFP‐Puro vector was used to overexpress the endogenous MLEC gene. ​​The wild‐type MLEC gene and MLEC mutant gene were synthesized and constructed by Gene Create (genecreate.cn, China). Subsequently, plasmids were co‐transfected with viral packaging plasmids psPAX2 (900 ng) and pMD2.G (100 ng; packaging vector : envelope vector = 1:9) into 293T cells, which was cultured in high glucose Dulbecco's modified Eagle medium (H‐DMEM) supplemented with 10% fetal calf serum, using Lipofectamine 2000 (Invitrogen; Thermo Fisher Scientific, Inc.) for 48 h at 37°C in a 5% CO2 incubator. Following 48 h of incubation, viral particles were collected, and then the viral supernatant (MOI = 10) was used to infect HTR‐8/SVneo and BeWo cells, in addition, polybrene reagent (5 µg/mL) was added to improve the transfection efficiency. MLEC gene and protein levels were then analyzed by qPCR and Western blot analysis (detailed below).

### 4.4 RT‑qPCR Assay

The RT‑qPCR assay was performed according to our previous procedure [61]. Total RNA was extracted from cells using TRIzol (Invitrogen) and reversely transcribed into cDNA using the HiScript II first Strand cDNA Synthesis Kit (Vazyme). Subsequently, qPCR was performed using the ChamQ SYBR qPCR Master Mix (Vazyme), and the data collection was performed on the StepOnePlus Real‐Time PCR Systems according to the manufacturer's instructions. The following thermocycling conditions were used for qPCR: Initial denaturation at 94°C for 5 min; and 40 cycles of 95°C for 5 s, 65°C for 34 s and 72°C for 30 s. The primer sequences for qPCR are shown in Table S9. The relative expression level of indicated genes was compared with that of β‐actin, and expression fold changes were calculated sing 2^−ΔΔCt^ methods. Experiments were performed in triplicate for each data point.

### 4.5 Western Blot Assay

Western blot analysis was performed following a previously established protocol [62]. HTR‐8/SVneo and BeWo cells were lysed in RIPA buffer (50 mm Tris‐acetate, pH 7.4; 0.5% Triton X‐100; 150 mm NaCl; 0.1 mm PMSF; protease inhibitor cocktail; 2 mm Na_3_VO_4_) on ice for 30 min. Placental tissues were homogenized in lysis buffer (Beyotime Biotechnology) under identical conditions, followed by centrifugation at 12 000 × g for 10 min at 4°C. Supernatants were collected, and protein concentrations were quantified using a BCA Protein Assay Kit (Beyotime). Proteins (20 µg per lane) were separated by sodium dodecyl sulfate‐polyacrylamide gel electrophoresis (SDS‐PAGE, 12% acrylamide) and transferred to nitrocellulose membranes (Cytiva). Membranes were blocked with 5% (w/v) nonfat dry milk in 1 × PBST (PBS containing 0.05% Tween‐20) for 1 h at room temperature, then incubated overnight at 4°C with primary antibodies targeting: malectin (Cat. No. 26655‐1‐AP; Proteintech), CHOP (Cat. No. AF6277; Affinity), GRP78 (Cat. No. M1506‐2; HuaBio), ATF‐4 (Cat. No. DF6008; Affinity), ATF‐6 (Cat. No. HA601321; HuaBio), p‐PERK (Cat. No. AF4499; Affinity), PERK (Cat. No. HA721510; HuaBio), Active Caspase‐3 (Cat. No. ET1602‐47; HuaBio), IRE1α (Cat. No. DF7709; Affinity), and p‐IRE1α (Cat. No. HA721980; HuaBio), OVOL1 (Cat. No. AY3490; Abways), Syncytin‐1 (Cat. No. 23172‐1‐AP; Proteintech), GLUT1 (Cat. No. ET1601‐10; HuaBio), HA‐Tag (Cat. No. B1003; Biodragon), Myc‐tag (Cat. No. B1002; Biodragon), α1‐Antitrypsin (Cat. No. ET1702‐87; HuaBio), VCP (Cat. No. CY6786; Abways), PI3K (Cat. No. AF6242; Affinity), AKT (Cat. No. ET1609‐47; HuaBio), GLUT4 (Cat. No. AF5386; Affinity). Following three washes with 1 × PBST, membranes were incubated with horseradish peroxidase (HRP)‐conjugated secondary antibodies (goat anti‐rabbit/mouse IgG) for 1 h at room temperature. Protein bands were visualized using SuperFemto ECL Master Mix (Vazyme) and the FluorChem E imaging system (ProteinSimple) according to manufacturer protocols. Densitometric analysis was performed using ImageJ 1.8.0 software (National Institutes of Health). Band intensities for target proteins were normalized to the corresponding Actin signals to control for loading variations. The normalized data were expressed as fold changes relative to the control group for statistical analysis.

### 4.6 High‐Throughput RNA Sequencing (RNA‐seq)

RNA‐seq was performed using Sh‐NC and Sh‐Mal HTR‐8/SVneo cells cultured to 80% confluence in 10 cm culture dishes. Cells were washed twice with phosphate‐buffered saline (PBS), lysed in 1 mL TRIzol reagent for 3 min, and transferred to 1.5 mL microcentrifuge tubes. Samples were stored on dry ice and shipped to Verygenome Technology (Guangzhou, China) for RNA‐seq library preparation and transcriptome analysis. Raw sequencing data were uploaded to the Verygenome Technology online platform (https://researcheasy.cn/tools) for differential gene expression analysis. Genes with fold Change ≥1.2 and adjusted *p* value ≤ 0.05 were identified as differentially expressed genes (DEGs). Functional enrichment of DEGs was conducted using Gene Ontology (GO) and Kyoto Encyclopedia of Genes and Genomes (KEGG) pathway analyses via the Verygenome Technology portal. Three biological triplicates were included for each experimental group to ensure statistical robustness. The data discussed in this publication have been deposited in NCBI's Gene Expression Omnibus [63] and are accessible through GEO Series accession number GSE294525.

### 4.7 Cell Apoptosis Assay

Cell apoptosis assays were conducted following a modified protocol from our prior study [64]. Annexin V‐FITC/PI and Annexin V‐kFluor647/PI apoptosis kits (KeyGEN BioTECH, China) were utilized to differentially label wild‐type and lentivirus‐infected HTR‐8/SVneo cells, respectively. Following 48 h treatment with 25 mm glucose, cells were washed twice with PBS and resuspended in binding buffer. The cells were subsequently stained with annexin V and PI for 15 min at room temperature in the dark. Following dilution with binding buffer, cell populations were analyzed using a BD FACSCanto II System (BD Biosciences) configured for two‐color analysis. The gating strategy involved sequential selection of intact cells based on FSC‐A/SSC‐A, exclusion of doublets using FSC‐H/FSC‐A, and quantification of apoptotic populations (Annexin V+/PI‐ for early apoptosis; Annexin V+/PI+ for late apoptosis/necrosis). Unstained and single‐stained controls for setting appropriate compensation and photomultiplier tube voltages. (Figure S10). All assays were independently repeated three times to ensure reproducibility.

### 4.8 Cell Invasion Assay

Cell invasion capacity was evaluated using Transwell chambers (Corning, USA). For the invasion assay, cells were exposed to high glucose for 48 h, and matrigel (Corning, USA) was evenly coated in the upper chamber and allowed to solidify. Subsequently, 1 × 10^5^ cells suspended in 200 µL of serum‐free medium were seeded in the upper chamber, and 500 µL of medium containing 10% FBS was added to the lower chamber. After incubating for 48 h, the Transwell chambers were fixed with methanol at room temperature for 30 min and stained with 0.2% crystal violet for 30 min. The cells on the upper surface were removed with a cotton swab, and invaded cells on the lower surface were observed and imaged under a microscope.

### 4.9 2‑NBDG Uptake Assay

Glucose uptake in BeWo cells was analyzed using a 2‐NBDG Glucose Uptake Assay Kit (Beyotime, China) combined with flow cytometry. Following a 48 h treatment with high glucose, the cells were stimulated with forskolin (FSK) at a final concentration of 20 µm for 24 h. Subsequently, the cells were harvested and incubated with 2‐NBDG fluorescent probe according to the manufacturer's protocol. Finally, cells were collected, and the intracellular fluorescence intensity was immediately measured using flow cytometry.

### 4.10 Preparation of Truncated Form of Malectin

Mal_42‐228 represents a truncated variant of malectin spanning amino acids 42 to 228. The coding sequence for Mal_42‐228 was synthetically engineered and subcloned into the pCold II expression vector by SynBio Technologies. The Mal_42‐228_6A variant was generated by introducing alanine substitutions at all six key carbohydrate‐binding residues of Mal_42‐228. To enable cellular delivery and ER retention, we engineered the TAT‐Mal (TAT‐2HA‐Mal_42‐228‐KDEL) protein by sequentially adding an N‐terminal transactivator of transcription (TAT) cell‐penetrating peptide and a dual HA tag, along with a C‐terminal KDEL signal, to the Mal_42‐228 core. Additionally, the TAT‐Mal_6A (TAT‐2HA‐Mal_42‐228_6A‐KDEL) construct was generated in a parallel manner. The coding sequences for both Mal_42‐228_6A, TAT‐Mal, and TAT‐Mal_6A were synthetically constructed and subcloned into the pCold II expression vector by GeneCreate (genecreate.cn). Recombinant protein expression was conducted in Escherichia coli BL21 (DE3) pLysS cells induced with 0.5 mm IPTG for 16 h at 25°C. Following affinity purification via Ni‐NTA agarose chromatography (Qiagen) according to previous studies [61, 64]. Mal_42‐228 and Mal_42‐228_6A were dialyzed against 10 mm Tris‐HCl (pH 7.5) and 150 mm NaCl, while TAT‐Mal and TAT‐Mal_6A were dialyzed in PBS. His‐tags were enzymatically removed using thrombin (5 units/mg protein). As determined by SDS‐PAGE, protein purity was >90% and stored at ‐80°C.

### 4.11 ITC Assay

The purified Malectin‐42‐228 was dialyzed overnight in a buffer containing 10 mm Tris, pH 7.5, 150 mm NaCl. Isothermal titration calorimetry (ITC) experiments were performed using a MicroCal PEAQ‐ITC system (Malvern Panalytical) at 25°C with a 0.1 mm concentration of Malectin‐42‐228 loaded into the sample cell, and 4 mm nigerose or maltose was injected into the cell by a given syringe. Each titration consists of 19 injections of 2 µL with 150 s intervals. The heat exchange by nigerose to buffer titration and maltose to buffer titration was also estimated, and the value was subtracted from the respective individual experiment. Binding isotherms were fitted into a one‐site binding model using the MicroCal PEAQ‐ITC analysis software.

### 4.12 Blue‐Native PAGE Assay

The oligomeric state of malectin was evaluated using the BeyoGel Blue Native‐PAGE system (Beyotime Biotechnology) according to the manufacturer's instructions. For the analysis of purified proteins, 3 µL of 4 × Native‐PAGE Sample Buffer was added to 10 µg (in 10 µL) of either Malectin‐42‐228 or Malectin‐42‐228_6A protein, and the mixtures were immediately loaded onto a 15% Native‐PAGE gel. An unstained protein standard (MeilunBio) was used as a molecular weight ladder. The entire electrophoresis procedure was performed on ice to preserve the integrity of the protein complexes. Upon completion, the gel was stained with Coomassie Brilliant Blue.

To examine the dimeric form of malectin expressed in eukaryotic cells, HTR‐8/SVneo cells were transfected with the recombinant plasmids pcDNA3.1‐Malectin‐Myc and pcDNA3.1‐Malectin_6A‐Myc, respectively. Freshly prepared cell lysates were collected. Similarly, a 12% Blue Native‐PAGE gel was prepared as instructed. A volume of 3 µL 4 × Native‐PAGE Sample Buffer was mixed with 10 µg (10 µL) of cell lysate, and the sample was then subjected to electrophoresis using the same method described for the purified Malectin‐42‐228 proteins. After electrophoresis, the lane containing the unstained native protein marker was excised and stained with Coomassie blue. The remaining gel strip was transferred onto a pre‐cut PVDF membrane with a 0.22 µm pore size. The transfer buffer was supplemented with SDS to a final concentration of 0.02%. Electroblotting was conducted at a constant voltage of 100 V for 60 min on ice.

Following transfer, the PVDF membrane was immersed in 100% methanol to remove the blue G‐250 dye, equilibrated in TBST for 5 min, and then incubated in TBST containing 0.5% reduced Triton X‐100 for 20 min. Subsequently, standard western blotting procedures were performed, including blocking, treatment with primary and secondary antibodies, washing, and final detection. Protein bands were visualized using a hypersensitive ECL chemiluminescence kit (Vazyme) and the FluorChem E imaging system (ProteinSimple).

### 4.13 Chemical Cross‐Linking Assay

Chemical cross‐linking assays were conducted following a modified protocol from our prior reported [65]. A total of 100 µg of Mal_42‐228 or Mal_42‐228‐6A were cross‐linked using 0.05% (v/v) glutaraldehyde in PBS, pH 7.4. After 30 min of incubation at 4°C, the reaction was stopped by addition of 100 mm Tris‐HCl. Mal_42‐228 or Mal_42‐228‐6A and their cross‐linked products were analyzed by SDS‐PAGE and visualized with Coomassie Blue Staining.

### 4.14 Carbohydrate‐Binding Activity Assay

This experiment was conducted following a standardized protocol and prior research [66]. Sepharose 6B matrices were conjugated to glucose, maltose, nigerose, and mannose, respectively. Binding assays were performed by incubating 100 µg of Mal_42‐228 or Mal_42‐228_6A with 10 µL of each Sepharose 6B conjugate in Eppendorf tubes at 20°C for 1 h. The bead‐bound complexes were washed four times with Tris buffer, followed by elution with 2 × SDS loading buffer preheated to 98°C for 10 min. Post‐elution supernatants were collected and resolved by SDS‐PAGE.

### 4.15 Circular Dichroism (CD)

The secondary structures of Mal_42‐228 and Mal_42‐228_6A were analyzed by CD spectroscopy using a JASCO J‐815 spectropolarimeter (Jasco Co., Tokyo, Japan), following a previously described procedure with modifications [61]. Proteins were diluted in PBS to a final concentration of 0.1 mg/mL. Far‐UV CD spectra were recorded from 200 to 260 nm using a cuvette with a 1 mm path length. After equilibration at 4°C for 2 h, spectra were acquired in continuous scanning mode at 50 nm/min. PBS was used as the background and subtracted from sample measurements. All experiments were performed in duplicate, and the percentages of secondary structure elements were quantified using the deconvolution software Bestsel [67].

### 4.16 TAT‐Mal Intracellular Analysis

Intracellular TAT‐Mal and TAT‐Mal_6A were assessed in parallel. HTR‐8/SVneo cells were treated with the respective proteins at final concentrations of 1, 5 and 10 µm for 5 h. Lysates were then analyzed by Western blot using an anti‐HA antibody.

### 4.17 Immunofluorescence

To visualize the intracellular localization of TAT‐Mal and TAT‐Mal_6A, HTR‐8/SVneo cells were incubated with the respective proteins for 5 h. After PBS washing to remove uninternalized protein, cells were fixed with 4% paraformaldehyde for 20 min at room temperature. After PBS washing, cells were permeabilized and blocked with 0.3% Triton X‐100 and 10% goat serum at 37°C for 60 min. The cells were then incubated overnight at 4°C with primary antibodies against Calnexin (Cat. No. BY2083; Abways) and HA‐tag (Cat. No. B1003; Biodragon) antibodies (diluted 1:200), followed by PBS‐T washing and a 1 h incubation with secondary antibodies at room temperature. Cell nuclei were counterstained with DAPI. The ER localization of overexpressed malectin and malectin_6A was also assessed in HTR‐8/Svneo and Bewo cells. Following a similar experimental procedure, the fixed and blocked cells were incubated overnight at 4°C with primary antibodies against malectin and Protein disulphide isomerase (DPI, Cat. No. 66422‐1‐Ig; Proteintech). After incubation with fluorescent secondary antibodies, the nuclei were stained with DAPI. Finally, the samples were mounted for imaging using a confocal fluorescence microscope (Zeiss LSM880, Germany).

### 4.18 Immunoprecipitation

To investigate the role of malectin in ER‐associated degradation (ERAD), we utilized AT^NHK^, a carboxyl terminus–truncated α1‐antitrypsin variant known to exhibit a defective folding phenotype, as a well‐established model ERAD substrate [30, 68]. The fusion genes encoding AT^NHK^‐HA, malectin‐Myc, and malectin_6A‐Myc were synthesized by Gene Create (genecreate.cn, China) and cloned into the pcDNA3.1 vector. HTR‐8/SVneo cells were divided into three experimental groups: Group 1 was co‐transfected with pcDNA3.1‐AT^NHK^‐HA and pcDNA3.1‐Myc; Group 2 with pcDNA3.1‐ pcDNA3.1‐AT^NHK^‐HA and pcDNA3.1‐malectin‐Myc; Group 3 with pcDNA3.1‐AT^NHK^‐HA and pcDNA3.1‐malectin_6A‐Myc. Forty‐eight hours post‐transfection, cells were lysed and the lysate was divided into four aliquots. One aliquot served as the input control; another was incubated with Protein A/G (Beyotime, China) as IgG control; a third was subjected to immunoprecipitation using Protein A/G coupled with an anti‐Myc antibody (Cat. No. B1002; Biodragon); and the final aliquot was used for Western blot analysis to assess the activation status of the ERAD marker p97/VCP (Cat. No. CY6786; Abways). Immune complexes from the co‐immunoprecipitation assay were analyzed by SDS‐PAGE under non‐reducing conditions (without DTT or β‐mercaptoethanol). Anti–α1‐antitrypsin antibody was purchased from HuaBio (Cat. No. ET1702‐87).

### 4.19 Crystallization, Data Collection, and Structure Determination of Mal_42‐228

Hampton Research packs (PEGRx 1, PEGRx 2, SaltRx 1, SaltRx 2, Index 1, Index 2, Crystal Screen 1, and Crystal Screen 2) were used for the initial crystallization screen (sitting‐drop vapor diffusion method). After incubated four days at 25°C, small crystals had formed under No. 23 Crystal Screen 2. Hanging‐drop and sitting‐drop methods were used to obtain a crystal that was suitable for x‐ray diffraction. Larger crystals were obtained from drops that contained 1 µL protein (15 mg/mL) and 1 µL solution from well containing 0.1 m MES pH 7.0–7.5, 1.9–2.1 m (NH4)2SO4 and 10% (W/V) 1,4‐Dioxane (hanging‐drop method) at 25°C. Glucose, maltose, nigerose and mannose with a final concentration of 5 mm were also added to the well solution for crystal growth. Prior to x‐ray data collection, crystals were soaked for approximately 1 min in reservoir solution supplemented with 20% (v/v) glycerol as a cryoprotectant, and then flash cooled in liquid nitrogen. All crystals were grown at the same temperature. Data sets were collected at 100 K at Shanghai Synchrotron Radiation Facility (Shanghai, China).

Data sets were indexed and integrated using AutoPX [69]. Structures were determined by Phaser with a molecular replacement method using structure model predicted by AlphaFold (AF‐Q14165‐F1) as search model [70]. Structure refinement and water updating were performed using Phenix refine and manual adjustment [71]. Final structure validations were performed using MolProbity [72, 73]. Figures for all structures were generated by using PyMOL.

### 4.20 Computational Modeling of the Malectin_6A Mutant Structure

The three‐dimensional structure of the malectin_6A mutant dimer was predicted using the automated homology modeling server SWISS‐MODEL [74]. The amino acid sequence of the mutant, which contains six alanine substitutions (S80A, E102A, Y104A, Y131A, K138A, D201A) within the wild‐type malectin sequence, was used as the target. The crystal structure of wild‐type human malectin dimer (PDB ID: 9IKP) was used as the primary template based on its high sequence identity (96.7%) to the target. The modeling process was performed in the “oligomeric” mode to generate a dimeric model. Structural analysis, superposition, and visualization were performed using the PyMOL.

### 4.21 Transmission Electron Microscope (TEM)

To visualize the ultrastructure of the ER, cells were seeded into 6‐well culture plates and incubated for 12 h prior to fixation. After washing with 0.1 m phosphate buffer (PB, pH 7.4), cells were fixed in 2.5% glutaraldehyde at 4°C overnight. Subsequent post‐fixation was performed using 1% osmium tetroxide at 4°C for 2 h, followed by dehydration through a graded ethanol series and embedding in Epoxy resin. Ultrathin sections (60–80 nm) were sequentially stained with 2% uranyl acetate and lead citrate, and were then observed using a transmission electron microscope (Hitachi HT7700).

### 4.22 Animals

All animal procedures were performed in accordance with the Guidelines for Care and Use of Laboratory Animals of Xuzhou Medical University and approved by the Animal Ethics Committee of Xuzhou Medical University (No. 202010A252). C57BL/6J mice (8 weeks old) were purchased from the GemPharmatech Co., Ltd and randomly assigned to 4 groups (n = 6 per group). Mice were housed in a stable facility with 12 h light/12 h dark cycles at 25°C. All mice were stabilized for a week to acclimatize to the experimental environment. The gestational day 0.5 (E0.5) was defined by the presence of a vaginal plug day. A GDM mouse model was established via a single intraperitoneal injection of streptozotocin (STZ, purchased from Yeasen Biotechnology (Shanghai) Co., Ltd.; 100 mg/kg, dissolved in 0.1 mmol/L citrate buffer, pH 4.2–4.5) after a 12 h fast at embryonic day 12.5 (E12.5) [75, 76]. The control mice (n = 6) were injected with the same amount of citrate buffer (CPBS). The GDM mice were then randomly assigned into four experimental groups, the first group receives only STZ (n = 6). The second group, STZ + TAT‐Mal‐1 (n = 6), was treated with the TAT‐Mal (2 mg/kg, dissolved in PBS), and administered intraperitoneally in E13.5‐E17.5. The third group, STZ + TAT‐Mal‐2 (n = 6), was treated with the TAT‐Mal (10 mg/kg, dissolved in PBS) following the same administration schedule. A fourth group, STZ + TAT‐Mal_6A (n = 6), was treated with the mutant TAT‐Mal_6A (10 mg/kg, dissolved in PBS) during the same gestational window (E13.5‐E17.5) to serve as a functional control. On E18.5, mice were anesthetized with an intraperitoneal injection of 3% pentobarbital sodium solution. Blood was collected by cardiac puncture and centrifuged at 3000 r/min for 15 min. The placenta tissues and vital organs (liver, spleen, lung, and kidney) were collected after sacrificing animals.

### 4.23 Glucose Tolerance Tests

For the glucose tolerance test (GTT), mice were fasted 16 h and then intraperitoneally injected with 2 g/kg body weight of glucose dissolved in saline on the 17th day of pregnancy. Blood glucose levels were then measured from tail vein blood with a glucometer and test strips at 0, 30, 60, 90, and 120 min after injection. The results of GTTs were displayed as blood glucose curves and the area under the curve (AUC).

### 4.24 Enzyme‑Linked Immunosorbent Assay (ELISA)

The ELISA assay was performed to examine the contents of insulin in serum samples. This analysis was conducted spectrophotometrically using diagnostic reagent kits and an enzyme‐linked immunosorbent assay kits sourced from Ruixinbio (Quanzhou, China).

### 4.25 Statistical Analysis

All data were processed and analyzed using GraphPad Prism 5.0 (GraphPad Software, La Jolla, CA, USA). Quantitative data are expressed as the mean ± standard deviation (SD). The sample size (n) for each experiment, representing biological replicates, is indicated in the corresponding figure legends. For comparisons between two groups, an unpaired two‐tailed Student's t‐test was used. For comparisons among multiple groups, one‐way analysis of variance (ANOVA) was performed, followed by Tukey's post hoc test for pairwise comparisons. A *p* value less than 0.05 was considered statistically significant.

## Author Contributions

Y.S. conceived and designed the study, analyzed the data, and wrote the manuscript. Y.L. provided guidance for the mouse experiments. J.Z. and Y.Z. performed the majority of the experiments and part of the data analysis. Y.W., X.Z., A.Y., W.Y., H.Y. and T.W. participated in partial experiments. X.P., Y.H., and Y.W. were responsible for placenta sample collection and processing. All authors reviewed the manuscript and provided critical feedback during its preparation.

## Funding

This work was supported by grants from National Natural Science Foundation of China (82201916), Postgraduate Research & Practice Innovation Program of Jiangsu Province (KYCX25_3219), Innovation and Entrepreneurship Training Program for College Students in Jiangsu Province (202410313109Y; S202510313071). National Experimental Demonstration Center For Basic Medicine Education (Xuzhou Medical University) Project (2025BMS26).

## Conflicts of Interest

The authors declare no conflicts of interest.

## Supporting information




**Supporting File 1**: advs75803‐sup‐0001‐SuppMat.docx.

**Supporting File 2**: advs75803‐sup‐0002‐Data.zip.

## Data Availability

The atomic coordinates and structure factors for human malectin, human malectin in complex with glucose, human malectin in complex with maltose and human malectin in complex with nigerose were deposited to the PDB under accession code 9IKP; 9IL3, 9ILA and 9ILF, respectively. The RNA‐seq data of Sh‐Con and Sh‐Mal have been deposited in NCBI's Gene Expression Omnibus and are accessible through GEO Series accession number GSE294525. All other data and materials are available from the corresponding authors upon request.

## References

[1] R. Retnakaran , C. Ye , A. J. Hanley , P. W. Connelly , M. Sermer , and B. Zinman , “Treatment of Gestational Diabetes Mellitus and Maternal Risk of Diabetes After Pregnancy,” Diabetes Care 46, no. 3 (2023): 587–592, 10.2337/dc22-1786. 36602334

[2] M. Lende and A. Rijhsinghani , “Gestational Diabetes: Overview With Emphasis on Medical Management,” International Journal of Environmental Research and Public Health 17, no. 24 (2020): 9573, 10.3390/ijerph17249573. 33371325 PMC7767324

[3] H. D. McIntyre , U. Kampmann , T. D. Clausen , J. Laurie , and R. C. W. Ma , “Gestational Diabetes: an Update 60 Years After O'Sullivan and Mahan,” The Journal of Clinical Endocrinology & Metabolism 110 (2024): e19–e31, 10.1210/clinem/dgae709. 39389786 PMC11651698

[4] E. C. Johns , F. C. Denison , J. E. Norman , and R. M. Reynolds , “Gestational Diabetes Mellitus: Mechanisms, Treatment, and Complications,” Trends in Endocrinology & Metabolism 29, no. 11 (2018): 743–754, 10.1016/j.tem.2018.09.004. 30297319

[5] A. Homayouni , N. Bagheri , S. Mohammad‐Alizadeh‐Charandabi , et al., “Prevention of Gestational Diabetes Mellitus (GDM) and Probiotics: Mechanism of Action: a Review,” Current Diabetes Reviews 16, no. 6 (2020): 538–545, 10.2174/1573399815666190712193828. 31544699

[6] J. W. Xie , L. Li , and H. Y. Xing , “Metabolomics in Gestational Diabetes Mellitus: A Review,” Clinica Chimica Acta 539 (2023): 134–143, 10.1016/j.cca.2022.12.005. 36529269

[7] P. Walter and D. Ron , “The Unfolded Protein Response: From Stress Pathway to Homeostatic Regulation,” Science 334, no. 6059 (2011): 1081–1086, 10.1126/science.1209038. 22116877

[8] A. Onn and D. Ron , “Modeling the Endoplasmic Reticulum Unfolded Protein Response,” Nature Structural & Molecular Biology 17, no. 8 (2010): 924–925, 10.1038/nsmb0810-924. 20683476

[9] H. Liao , S. Liu , Q. Ma , et al., “Endoplasmic Reticulum Stress Induced Autophagy in Cancer and its Potential Interactions With Apoptosis and Ferroptosis,” Biochimica et Biophysica Acta (BBA) ‐ Molecular Cell Research 1872, no. 1 (2025): 119869, 10.1016/j.bbamcr.2024.119869. 39490702

[10] E. Guzel , M. Basar , N. Ocak , A. Arici , and U. A. Kayisli , “Bidirectional Interaction between Unfolded‐Protein‐Response Key Protein HSPA5 and Estrogen Signaling in Human Endometrium1,” Biology of Reproduction 85, no. 1 (2011): 121–127, 10.1095/biolreprod.110.089532. 21389343

[11] G. Bifulco , C. Miele , B. Di Jeso , et al., “Endoplasmic Reticulum Stress is Activated in Endometrial Adenocarcinoma,” Gynecologic Oncology 125, no. 1 (2012): 220–225, 10.1016/j.ygyno.2011.11.045. 22146569

[12] E. Guzel , S. Arlier , O. Guzeloglu‐Kayisli , et al., “Endoplasmic Reticulum Stress and Homeostasis in Reproductive Physiology and Pathology,” International Journal of Molecular Sciences 18, no. 4 (2017): 792, 10.3390/ijms18040792. 28397763 PMC5412376

[13] M. Mizuuchi , T. Cindrova‐Davies , M. Olovsson , D. S. Charnock‐Jones , G. J. Burton , and H. W. Yung , “Placental Endoplasmic Reticulum Stress Negatively Regulates Transcription of Placental Growth Factor via ATF4 and ATF6β: Implications for the Pathophysiology of human Pregnancy Complications,” The Journal of Pathology 238, no. 4 (2016): 550–561, 10.1002/path.4678. 26648175 PMC4784173

[14] W. H. He , Y. T. Zhao , L. J. Yin , et al., “The Transcription Factor XBP1 Regulates Mitochondrial Remodel and Autophagy in Spontaneous Abortion,” International Immunopharmacology 152 (2025): 114398, 10.1016/j.intimp.2025.114398. 40068517

[15] Y. Zheng , X. Zha , B. Zhang , et al., “The Interaction of ER Stress and Autophagy in Trophoblasts: Navigating Pregnancy Outcome,” Biology of Reproduction 111, no. 2 (2024): 292–311, 10.1093/biolre/ioae066. 38678504

[16] Y. Jiang , Q. X. You , F. X. Mu , S. Q. Xiang , and N. Zhang , “Endoplasmic Reticulum Stress and Unfolded Protein Response Play Roles in Recurrent Pregnancy Loss: A Bioinformatics Study,” Journal of Reproductive Immunology 168 (2025): 104446, 10.1016/j.jri.2025.104446. 39923360

[17] L. Y. Qiu , H. Liu , S. L. Chen , Y. T. Wu , and J. Y. Yan , “Ferroptosis Contributed to Endoplasmic Reticulum Stress in Preterm Birth by Targeting LHX1 and IRE‐1,” Cellular Signalling 132 (2025): 111777, 10.1016/j.cellsig.2025.111777. 40157471

[18] H.‐W. Yung , P. Alnæs‐Katjavivi , C. J. P. Jones , et al., “Placental Endoplasmic Reticulum Stress in Gestational Diabetes: the Potential for Therapeutic Intervention with Chemical Chaperones and Antioxidants,” Diabetologia 59, no. 10 (2016): 2240–2250, 10.1007/s00125-016-4040-2. 27406815 PMC5016560

[19] M. He , X. Guo , J. Jia , et al., “Regulatory Mechanisms Underlying Endoplasmic Reticulum Stress Involvement in the Development of Gestational Diabetes Mellitus Entail the CHOP‐PPARα–NF–κB Pathway,” Placenta 142 (2023): 46–55, 10.1016/j.placenta.2023.08.070. 37639950

[20] J. Torres‐Torres , I. E. Monroy‐Muñoz , J. Perez‐Duran , et al., “Cellular and Molecular Pathophysiology of Gestational Diabetes,” International Journal of Molecular Sciences 25, no. 21 (2024): 11641, 10.3390/ijms252111641. 39519193 PMC11546748

[21] S. Sarikaya , E. Almaghrebi , F. Akat , et al., “Investigation of Endoplasmic Reticulum Stress Parameters in Patients with Gestational Diabetes Mellitus: a Prospective Study,” International Journal of Gynecology & Obstetrics 170, no. 2 (2025): 774–782, 10.1002/ijgo.70091. 40145401 PMC12255919

[22] Q. Wang , J. Groenendyk , and M. Michalak , “Glycoprotein Quality Control and Endoplasmic Reticulum Stress,” Molecules 20, no. 8 (2015): 13689–13704, 10.3390/molecules200813689. 26225950 PMC6331979

[23] S. P. Ferris , V. K. Kodali , and R. J. Kaufman , “Glycoprotein Folding and Quality‐Control Mechanisms in Protein‐Folding Diseases,” Disease Models & Mechanisms 7, no. 3 (2014): 331–341, 10.1242/dmm.014589. 24609034 PMC3944493

[24] T. Keser , I. Gornik , F. Vuckovic , et al., “Increased Plasma N‐glycome Complexity is Associated With Higher Risk of Type 2 Diabetes,” Diabetologia 60, no. 12 (2017): 2352–2360, 10.1007/s00125-017-4426-9. 28905229

[25] A. Wahl , E. van den Akker , L. Klaric , et al., “Genome‐Wide Association Study on Immunoglobulin G Glycosylation Patterns,” Frontiers in Immunology 9 (2018): 277, 10.3389/fimmu.2018.00277. 29535710 PMC5834439

[26] K. Ohtsubo , S. Takamatsu , C. Gao , H. Korekane , T. M. Kurosawa , and N. Taniguchi , “Glycosylation Modulates the Membrane Sub‐domain Distribution and Activity of Glucose Transporter 2 in Pancreatic Beta Cells,” Biochemical and Biophysical Research Communications 434, no. 2 (2013): 346, 10.1016/j.bbrc.2013.03.076. 23548572

[27] C. L. Lee , P. C. N. Chiu , P. C. Pang , et al., “Glycosylation Failure Extends to Glycoproteins in Gestational Diabetes Mellitus Evidence From Reduced α2‐6 Sialylation and Impaired Immunomodulatory Activities of Pregnancy‐Related Glycodelin‐A,” Diabetes 60, no. 3 (2011): 909, 10.2337/db10-1186. 21300843 PMC3046852

[28] T. Zhou , L. Huang , M. Wang , D. Z. Chen , Z. Chen , and S. W. Jiang , “A Critical Review of Proteomic Studies in Gestational Diabetes Mellitus,” Journal of Diabetes Research 2020 (2020): 1–13, 10.1155/2020/6450352. PMC7381988 32724825

[29] T. Schallus , C. Jaeckh , K. Fehér , et al., “Malectin: a Novel Carbohydrate‐Binding Protein of the Endoplasmic Reticulum and a Candidate Player in the Early Steps of Protein N ‐Glycosylation,” Molecular Biology of the Cell 19, no. 8 (2008): 3404–3414, 10.1091/mbc.E08-04-0354. 18524852 PMC2488313

[30] Y. Chen , D. Hu , R. Yabe , et al., “Role of Malectin in Glc_2_Man_9_GlcNAc_2_‐Dependent Quality Control of α1‐antitrypsin,” Molecular Biology of the Cell 22, no. 19 (2011): 3559, 10.1091/mbc.E11-03-0201. 21813736 PMC3183012

[31] C. Galli , R. Bernasconi , T. Soldà , V. Calanca , and M. Molinari , “Malectin Participates in a Backup Glycoprotein Quality Control Pathway in the Mammalian ER,” PLoS ONE 6, no. 1 (2011): 16304, 10.1371/journal.pone.0016304. PMC3027649 21298103

[32] Y. Ban , G. Yamamoto , M. Takada , et al., “Proteomic Profiling of Thyroid Papillary Carcinoma,” Journal of Thyroid Research 2012 (2012): 1–7, 10.1155/2012/815079. PMC3306988 22518348

[33] S. Sugiura , H. Yoshida , H. Sugiura , et al., “Increased Intracellular Stress Responses and Decreased KLF2 in Adult Patients With Atopic Dermatitis,” Cell Stress and Chaperones 30, no. 2 (2025): 84–99, 10.1016/j.cstres.2025.02.001. 39938773 PMC11891603

[34] Y. Dong , M. F. Fu , S. M. Liu , et al., “Malectin, an Endoplasmic Reticulum‐Resident Lectin, Promotes Malignant Behavior of Human Hepatocellular Carcinoma,” Glycobiology 35, no. 4 (2025): cwaf007, 10.1093/glycob/cwaf007. 39987555

[35] N. Jiang , Y. F. Xiao , Y. S. Liu , W. H. Liu , and S. X. Liu , “Blood Coagulation Factor VIII D1241E Polymorphism Leads to a Weak Malectin Interaction and Reduction of Factor VIII Posttranslational Modification and Secretion,” Experimental Cell Research 397, no. 1 (2020): 112334, 10.1016/j.yexcr.2020.112334. 33144078

[36] W. Shi , Y. Zhu , M. Zhou , Y. Ruan , X. Chen , and X. Chen , “Malectin Gene Polymorphisms Promote Cerebral Palsy via M2‐Like Macrophage Polarization,” Clinical Genetics 93, no. 4 (2018): 794–799, 10.1111/cge.13149. 28972276

[37] J. Pei , Y. Liao , X. Bai , et al., “Dysregulated GLUT1 Results in the Pathogenesis of Preeclampsia by Impairing the Function of Trophoblast Cells,” Scientific Reports 14, no. 1 (2024): 23761, 10.1038/s41598-024-74489-z. 39390043 PMC11467397

[38] J. A. Choi and C. H. Song , “Insights Into the Role of Endoplasmic Reticulum Stress in Infectious Diseases,” Frontiers in Immunology 10 (2020): 3147, 10.3389/fimmu.2019.03147. 32082307 PMC7005066

[39] M. M. Shi , Y. Chai , J. N. Zhang , and X. Chen , “Endoplasmic Reticulum Stress‐Associated Neuronal Death and Innate Immune Response in Neurological Diseases,” Frontiers in Immunology 12 (2022): 794580, 10.3389/fimmu.2021.794580. 35082783 PMC8784382

[40] U. Özcan , Q. Cao , E. Yilmaz , et al., “Endoplasmic Reticulum Stress Links Obesity, Insulin Action, and Type 2 Diabetes,” Science 306, no. 5695 (2004): 457, 10.1126/science.1103160. 15486293

[41] B. Schisano , A. L. Harte , K. Lois , et al., “GLP‐1 Analogue, Liraglutide Protects Human Umbilical Vein Endothelial Cells Against High Glucose Induced Endoplasmic Reticulum Stress,” Regulatory Peptides 174, no. 1 (2012): 46–52, 10.1016/j.regpep.2011.11.008. 22120833

[42] A. K. Cardozo , F. Ortis , J. Storling , et al., “Cytokines Downregulate the Sarcoendoplasmic Reticulum Pump Ca^2+^ ATPase 2b and Deplete Endoplasmic Reticulum Ca^2+^, Leading to Induction of Endoplasmic Reticulum Stress in Pancreatic β‐Cells,” Diabetes 54, no. 2 (2005): 452–461, 10.2337/diabetes.54.2.452. 15677503

[43] Y.‐Y. Chen , X.‐F. Peng , G.‐Y. Liu , et al., “Protein Arginine Methyltranferase‐1 Induces ER Stress and Epithelial‐mesenchymal Transition in Renal Tubular Epithelial Cells and Contributes to Diabetic Nephropathy,” Biochimica et Biophysica Acta (BBA) ‐ Molecular Basis of Disease 1865, no. 10 (2019): 2563–2575, 10.1016/j.bbadis.2019.06.001. 31199999

[44] S. Wilbur , D. Jones , J. F. Risher , et al., Toxicological Profile for 1,4‐Dioxane (Agency for Toxic Substances and Disease Registry (US), 2012). 23946965

[45] W. Quaglia , A. Piergentili , F. Del Bello , et al., “Structure−Activity Relationships in 1,4‐Benzodioxan‐Related Compounds. 9. (1) From 1,4‐Benzodioxane to 1,4‐Dioxane Ring as a Promising Template of Novel α_1D_‐Adrenoreceptor Antagonists, 5‐HT_1A_ Full Agonists, and Cytotoxic Agents†,” Journal of Medicinal Chemistry 51, no. 20 (2008): 6359–6370, 10.1021/jm800461k. 18817363

[46] F. Del Bello , A. Bonifazi , G. Giorgioni , et al., “Chemical Manipulations on the 1,4‐dioxane Ring of 5‐HT1A Receptor Agonists Lead to Antagonists Endowed with Antitumor Activity in Prostate Cancer Cells,” European Journal of Medicinal Chemistry 168 (2019): 461–473, 10.1016/j.ejmech.2019.02.056. 30844609

[47] F. Del Bello , E. Barocelli , S. Bertoni , et al., “1,4‐Dioxane, a Suitable Scaffold for the Development of Novel M_3_ Muscarinic Receptor Antagonists,” Journal of Medicinal Chemistry 55, no. 4 (2012): 1783–1787, 10.1021/jm2013216. 22243489

[48] F. A. Ortiz‐Morea , J. Liu , L. B. Shan , and P. He , “Malectin‐like Receptor Kinases as Protector Deities in Plant Immunity,” Nature Plants 8, no. 1 (2022): 27–37, 10.1038/s41477-021-01028-3. 34931075 PMC9059209

[49] C. M. Franck , J. Westermann , A. Boisson‐Dernier , et al., “Plant Malectin‐Like Receptor Kinases: From Cell Wall Integrity to Immunity and beyond,” Annual Review of Plant Biology 69 (2018): 301–328, 10.1146/annurev-arplant-042817-040557. 29539271

[50] S. Du , L. J. Qu , and J. Y. Xiao , “Crystal Structures of the Extracellular Domains of the CrRLK1L Receptor‐like Kinases ANXUR1 and ANXUR2,” Protein Science 27, no. 4 (2018): 886–892, 10.1002/pro.3381. 29388293 PMC5866931

[51] S. Moussu , S. Augustin , A. O. Roman , C. Broyart , and J. Santiago , “Crystal Structures of Two Tandem Malectin‐Like Receptor Kinases Involved in Plant Reproduction,” Acta Crystallographica Section D Structural Biology 74 (2018): 671–680, 10.1107/S205979831800774x. 29968676 PMC6038381

[52] A. Varki , R. D. Cummings , J. D. Esko , et al., Essentials of Glycobiology, 3rd ed. (Cold Spring Harbor Laboratory Press, 2015). 20301239

[53] P. Ulrich and A. Cerami , “Protein Glycation, Diabetes, and Aging,” Recent Progress in Hormone Research 56 (2001): 1–22, 10.1210/rp.56.1.1. 11237208

[54] D. C. W. Poland , C. G. Schalkwijk , C. D. A. Stehouwer , C. A. M. Koeleman , B. van het Hof , and W. van Dijk , “Increased α3‐fucosylation of α1‐acid Glycoprotein in Type I Diabetic Patients Is Related to Vascular Function,” Glycoconjugate Journal 18, no. 3 (2001): 261–268, 10.1023/A:1012412908983. 11602810

[55] Y. Deng , H. Y. Jin , J. Ning , D. Cui , M. Q. Zhang , and H. X. Yang , “Elevated Galectin‐3 Levels Detected in Women With Hyperglycemia During Early and Mid‐Pregnancy Antagonizes High Glucose − Induced Trophoblast Cells Apoptosis via Galectin‐3/foxc1 Pathway,” Molecular Medicine 29, no. 1 (2023): 115, 10.1186/s10020-023-00707-5. 37626284 PMC10463409

[56] H. Y. Peng , M. Q. Li , and H. P. Li , “High Glucose Suppresses the Viability and Proliferation of HTR‑8/SVneo Cells through Regulation of the miR‑137/PRKAA1/IL‑6 Axis,” International Journal of Molecular Medicine 42, no. 2 (2018): 799–810, 10.3892/ijmm.2018.3686. 29786111 PMC6034938

[57] G. H. Zeng , W. Y. Zou , C. D. Liu , Y. L. Chen , and T. M. Wen , “Ginsenoside Re Suppresses High Glucose‐induced Apoptosis of Placental Trophoblasts through Endoplasmic Reticulum Stress‐Related CHOP/GADD153,” Human & Experimental Toxicology 44 (2025): 09603271241307835, 10.1177/09603271241307835. 39798073

[58] M. Lytrivi , Y. Tong , E. Virgilio , X. Y. Yi , and M. Cnop , “Diabetes Mellitus and the Key Role of Endoplasmic Reticulum Stress in Pancreatic β Cells,” Nature Reviews Endocrinology 21, no. 9 (2025): 546–563, 10.1038/s41574-025-01129-5. 40467970

[59] J. H. Lee and J. Lee , “Endoplasmic Reticulum (ER) Stress and Its Role in Pancreatic β‐Cell Dysfunction and Senescence in Type 2 Diabetes,” International Journal of Molecular Sciences 23, no. 9 (2022): 4843, 10.3390/ijms23094843. 35563231 PMC9104816

[60] Z. H. Cao , Z. Wu , C. Hu , M. Zhang , W. Z. Wang , and X. B. Hu , “Endoplasmic Reticulum Stress and Destruction of Pancreatic β Cells in Type 1 Diabetes,” Chinese Medical Journal 133, no. 1 (2020): 68–73, 10.1097/Cm9.0000000000000583. 31923106 PMC7028193

[61] Y. Liang , Y. Wang , X. Zhu , et al., “Binding of Glycerol to Human Galectin‐7 Expands Stability and Modulates its Functions,” International Journal of Molecular Sciences 23, no. 20 (2022): 12318, 10.3390/ijms232012318. 36293173 PMC9604435

[62] Y. Si , Y. Li , T. Yang , et al., “Structure–function Studies of Galectin‐14, an Important Effector Molecule in Embryology,” The FEBS Journal 288, no. 3 (2021): 1041–1055, 10.1111/febs.15441. 32525264

[63] R. Edgar , M. Domrachev , and A. E. Lash , “Gene Expression Omnibus: NCBI Gene Expression and Hybridization Array Data Repository,” Nucleic Acids Research 30, no. 1 (2002): 207–210, 10.1093/nar/30.1.207. 11752295 PMC99122

[64] Y. L. Si , J. Cai , J. H. Zhu , et al., “Linker Remodels human Galectin‐8 Structure and Regulates its Hemagglutination and Pro‐apoptotic Activity,” International Journal of Biological Macromolecules 245 (2023): 125456, 10.1016/j.ijbiomac.2023.125456. 37331541

[65] Y. Si , S. Feng , J. Gao , et al., “Human Galectin‐2 Interacts with Carbohydrates and Peptides Non‐classically: New Insight From X‐ray Crystallography and Hemagglutination,” Acta Biochimica Et Biophysica Sinica 48, no. 10 (2016): 939–947, 10.1093/abbs/gmw089. 27563008

[66] J. Su , J. Gao , Y. Si , et al., “Galectin‐10: A New Structural Type of Prototype Galectin Dimer and Effects on Saccharide Ligand Binding,” Glycobiology 28, no. 3 (2018): 159–168, 10.1093/glycob/cwx107. 29293962

[67] A. Micsonai , F. Wien , L. Kernya , et al., “Accurate Secondary Structure Prediction and Fold Recognition for Circular Dichroism Spectroscopy,” Proceedings of the National Academy of Sciences of the United States of America 112, no. 24 (2015): E3095, 10.1073/pnas.1500851112. 26038575 PMC4475991

[68] N. Hosokawa , L. O. Tremblay , Z. P. You , A. Herscovics , I. Wada , and K. Nagata , “Enhancement of Endoplasmic Reticulum (ER) Degradation of Misfolded Null Hong Kong α1‐Antitrypsin by Human ER Mannosidase I,” Journal of Biological Chemistry 278, no. 28 (2003): 26287–26294, 10.1074/jbc.M303395200. 12736254

[69] L. Y. Wang , Y. H. Yun , Z. L. Zhu , and L. W. Niu , “AutoPX: A New Software Package to Process X‐ray Diffraction Data From Biomacromolecular Crystals,” Acta Crystallographica Section D Structural Biology 78 (2022): 890–902, 10.1107/S2059798322005745. 35775988

[70] A. J. McCoy , R. W. Grosse‐Kunstleve , P. D. Adams , M. D. Winn , L. C. Storoni , and R. J. Read , “Phaser Crystallographic Software,” Journal of Applied Crystallography 40 (2007): 658–674, 10.1107/S0021889807021206. 19461840 PMC2483472

[71] P. D. Adams , P. V. Afonine , G. Bunkóczi , et al., “PHENIX: A Comprehensive Python‐Based System for Macromolecular Structure Solution,” Acta Crystallographica Section D Biological Crystallography 66 (2010): 213–221, 10.1107/S0907444909052925. 20124702 PMC2815670

[72] I. W. Davis , A. Leaver‐Fay , V. B. Chen , et al., “MolProbity: All‐Atom Contacts and Structure Validation for Proteins and Nucleic Acids,” Nucleic Acids Research 35 (2007): W375–W383, 10.1093/nar/gkm216. 17452350 PMC1933162

[73] V. B. Chen , W. B. Arendall , J. J. Headd , et al., “MolProbity: All‐Atom Structure Validation for Macromolecular Crystallography,” Acta Crystallographica Section D Biological Crystallography 66 (2010): 12–21, 10.1107/S0907444909042073. 20057044 PMC2803126

[74] A. Waterhouse , M. Bertoni , S. Bienert , et al., “SWISS‐MODEL: Homology Modelling of Protein Structures and Complexes,” Nucleic Acids Research 46 (2018): W296–W303, 10.1093/nar/gky427. 29788355 PMC6030848

[75] Q. Zhang , X. Yuan , X. Luan , et al., “GLUT1 Exacerbates Trophoblast Ferroptosis by Modulating AMPK/ACC Mediated Lipid Metabolism and Promotes Gestational Diabetes Mellitus Associated Fetal Growth Restriction,” Molecular Medicine 30, no. 1 (2024): 257, 10.1186/s10020-024-01028-x. 39707215 PMC11660491

[76] Z. H. Xiao , X. Liu , X. J. Luan , et al., “Glucose Uptake in Trophoblasts of GDM Mice is Regulated by the AMPK‐CLUT3 Signaling Pathway,” Scientific Reports 14, no. 1 (2024): 12051, 10.1038/s41598-024-61719-7. 38802412 PMC11130200

