# Supplementary material for: Malectin Alleviates Endoplasmic Reticulum Stress in Gestational Diabetes Mellitus via Glycoprotein Quality Control Mechanisms
Source: Adv Sci (Weinh). 2026 May 25;13(46):e08901. doi: 10.1002/advs.202508901 (PMC13335976; doi:10.1002/advs.202508901)
Supplement: Supplementary file 1 [file ADVS-13-e08901-s002.docx]

Supporting Information

Malectin Alleviates Endoplasmic Reticulum Stress in Gestational Diabetes Mellitus via Glycoprotein Quality Control Mechanisms

Jiahui Zhu, Yumeng Zhang, Ye Wang, Xiaoyu Zhu, Ailin Yuan, Weijing Yin, Huangmin Yu, Xuemin Pang, Yufeng He, Yuchen Wang, Tong Wang, Yong Li*, Yunlong Si*.

Supplementary Table S1. Clinical characteristics of the validated subjects.

| Index | Normal (n=10) | GDM (n=10) | *P* |
| --- | --- | --- | --- |
| Age (years) | 30.7 ± 3.057 | 32.3 ± 3.917 | 0.322 |
| Gestational age of delivery (weeks) | 39.74 ± 1.007 | 39.31 ± 0.9916 | 0.3504 |
| Birth weight of newborn（kg） | 3.545 ± 0.3235 | 3.59 ± 0.4131 | 0.7839 |
| Nature conceived | Y | Y |  |
| Singleton pregnancy | Y | Y |  |
| Ethnicity | Han | Han |  |
| Second trimester BMI (kg/m^2^) | 24.45 ± 1.119 | 28.28 ± 3.821 | 0.0071 |
| Third trimester BMI (kg/m^2^) | 28.23 ± 2.777 | 30.16 ± 3.786 | 0.2096 |
| Fasting Blood Glucose (mmol/L) | 4.446 ± 0.3273 | 5.353 ± 0.6871 | 0.0014 |
| OGTT-1h (mmol/L) | 7.36 ± 1.123 | 10.03 ± 1.598 | 0.0004 |
| OGTT-2h (mmol/L) | 6.02 ± 1.142 | 9.36 ± 1.563 | <0.0001 |
| Ultrasound abnormality | N | N |  |
| Medical diseases | N | N |  |
| Family genetic history | N | N |  |

All quantitative data are presented as mean ± SD. Statistical significance was determined by unpaired two-tailed Student’s t-test. A P value less than 0.05 was considered statistically significant. BMI, body mass index; OGTT, oral glucose tolerance test; Y: Yes; N: No.

Supplementary Figure S2. Glucose gradient treatment and antibody validation for ER stress analysis. (A) Inhibition of the PI3K/AKT/GLUT4 signaling pathway under increasing glucose concentrations indicates impaired insulin signaling and diminished glucose uptake. (band intensities normalized to Actin; n=3) (B) Concurrent upregulation of Malectin suggests its role as a key adaptive factor against metabolic stress. (band intensities normalized to Actin; n=3) (C) A dose-dependent increase in apoptosis reflects the direct cytotoxicity of the high-glucose environment. (D) Tunicamycin, used as a positive control, activates all three UPR branches (PERK, IRE1α, ATF-6), validating the antibody specificity. (band intensities normalized to Actin; n=3). All quantitative data are presented as mean ± SD. Statistical significance was determined by unpaired two-tailed Student’s t-test or one-way ANOVA with Tukey’s post hoc test (****P < 0.0001, ***P< 0.001, **P < 0.01, *P < 0.05, ns > 0.05).

Supplementary Figure S3. Validation of Malectin knockdown specificity and functional assessment. (A, B) Knockdown efficiency was confirmed by Western blot (normalized to Actin) and qPCR (normalized to Actin). (C) Apoptosis was quantified by flow cytometry using Annexin V/PI staining (n=3), with gating on intact single cells. (D) Activation of ER stress pathways was evaluated by Western blot (normalized to Actin; n=3). (E) Cell invasion capacity was measured by Transwell assay, with cell counts from five independent fields per group (n=5). All quantitative data are presented as mean ± SD. Statistical significance was determined by one-way ANOVA with Tukey’s post hoc test (****P < 0.0001, ***P< 0.001, **P < 0.01, *P < 0.05, ns > 0.05).

Supplementary Table S4. Data collection and refinement statistics.

| **PDB code** | **9IKP** | **9IL3** | **9ILA** | **9ILF** |
| --- | --- | --- | --- | --- |
| Resolution (Å) | 33.42-1.68  (1.72-1.68) | 33.27-1.45  (1.49-1.45) | 33.02-1.56  (1.60-1.56) | 33.26-1.56  (1.60-1.56) |
| Space group | P31 | P31 | P31 | P31 |
| Unit cell parameters (a, b, c) (Å), (α, β, γ) (°) | (100.328,100.328,36.052)  (90.00,90.00,120.00) | (100.238,100.238,35.886)  (90.00,90.00,120.00) | (99.682,99.682,35.535)  (90.00,90.00,120.00) | (99.964,99.964,35.881)  (90.00,90.00,120.00) |
| No. of total observations | 470758 (35182) | 700693 (50004) | 552319 (39848) | 562501 (40945) |
| No. of unique observations | 46325 (3490) | 71579 (5309) | 56242 (4139) | 57060 (4148) |
| Completeness (%) | 100 (100) | 100 (100) | 100 (100) | 100 (100) |
| Multiplicity | 10.16 (10.08) | 9.79 (9.42) | 9.82 (9.63) | 9.86 (9.87) |
| <I/δ(I)> | 32.41 (10.79) | 29.04 (5.78) | 32.85 (10.25) | 40.33 (7.53) |
| Rmodel (%) | 16.92 | 17.52 | 17.07 | 17.66 |
| Rfree (%) | 20.74 | 19.64 | 18.31 | 19.68 |
| Rmsd bond lengths (Å) | 0.01 | 0.03 | 0.01 | 0..01 |
| Rmsd bond angles (°) | 1.17 | 2.18 | 1.07 | 1.13 |
| Ramachandran plotf residues in  favored regions (%) | 96.43 | 95.88 | 96.70 | 96.15 |
| Ramachandran outliers (%) | 0 | 0 | 0 | 0 |
| ligands | - | Glucose | Maltose | Nigerose |

Supplementary Figure S5. Functional characterization of human malectin: carbohydrate-binding activity, chemical cross-linking assay and localization of 1,4-dioxane molecules in human malectin structures. (A) SDS-PAGE analysis of carbohydrate-binding activities of malectin. (B) SDS-PAGE analysis of carbohydrate-binding activities of malectin_6A. (C) Chemical cross-linking assays of malectin and malectin_6A. (D, E and F) The 1,4-dioxane molecule is bound at the dimer interface between monomers A and B in the apo-, maltose-bound and nigerose-bound structures.

Supplementary Figure S6. Computational modeling confirms the preserved dimeric structure and altered surface electrostatics of malectin_6A. (A) Cartoon representation of the predicted dimeric structure of malectin_6A, generated using SWISS-MODEL, displaying all 14 β-strands and 5 α-helices per monomer. (B) Structural superposition of the malectin_6A dimer (orange sheet) onto the wild-type malectin crystal structure (magenta sheet) reveals a nearly identical fold with a backbone Cα RMSD of 0.057 Å. (C) Inter-monomer interaction network within the wild-type malectin dimer, showing 11 hydrogen bonds (yellow dashed lines) between Monomer A (green sticks) and Monomer B (red sticks), with distances labeled in angstroms (Å). (D) Equivalent inter-monomer interaction network within the malectin_6A mutant dimer, also exhibiting 11 hydrogen bonds (yellow dashed lines) with comparable bond lengths to the wild-type. (E) Close-up view highlighting the location of the six alanine mutation sites (cyan sticks) within the dimer interface, demonstrating that these mutations do not disrupt the key inter-monomer interactions. (F) Electrostatic surface potential map of wild-type malectin, calculated over the molecular surface with a potential range of -63.66 to +63.66 kT/e (red = negative, blue = positive); black circles denote the six carbohydrate-binding sites. (G) Electrostatic surface potential map of the malectin_6A mutant, showing a narrowed potential range of -59.44 to +59.44 kT/e; black circles highlight the regions corresponding to the six alanine substitution sites, indicating localized alterations in surface charge distribution.

Supplementary Figure S7. Immunofluorescence analysis showing ER localization of overexpressed malectin. (A) Co-localization of overexpressed malectin (grey) with the ER marker PDI (red) in HTR-8/Svneo cells. (B) Co-localization of overexpressed malectin_6A (grey) with the ER marker PDI (red) in HTR-8/Svneo cells. (C) Co-localization of overexpressed malectin (grey) with the ER marker PDI (red) in BeWo cells. (D) Co-localization of overexpressed malectin_6A (grey) with the ER marker PDI (red) in BeWo cells. The arrows indicate presence of overexpressed malectin and PDI in the nuclear envelope. Bar, 5 µm.

Supplementary Figure S8. Intracellular entry and tissue distribution of TAT-Mal. (A) Schematic diagram of the TAT-Mal and TAT-Mal_6A fusion proteins. (B) SDS-PAGE analysis demonstrating the purity of the recombinant TAT-Mal protein. (C) Western blot analysis of TAT-Mal internalization in HTR-8/SVneo cells after incubation via anti-HA-tag. (D) Immunofluorescence analysis showing co-localization of internalized TAT-Mal (green) with the ER marker Calnexin (red). The arrows indicate presence of TAT-Mal and Calnexin in the nuclear envelope. Bar, 10 µm. (E) SDS-PAGE analysis demonstrating the purity of the recombinant TAT-Mal_6A protein. (F) Western blot analysis of TAT-Mal_6A internalization in HTR-8/SVneo cells after incubation via anti-HA-tag. (G) Immunofluorescence analysis showing co-localization of internalized TAT-Mal_6A (green) with the ER marker Calnexin (red). The arrows indicate presence of TAT-Mal and Calnexin in the nuclear envelope. Bar, 10 µm. (H) Western blot analysis of TAT-Mal distribution in mouse tissues following intraperitoneal injection (normalized to Actin; n=3).

Supplementary Table S9. Primer sequences for qPCR

| Genes | Direction | Primer Sequences (5'-3') | Product length (bp) |
| --- | --- | --- | --- |
| ATF-4 | Forward | ATGACCGAAATGAGCTTCCTG | 153 |
|  | Reverse | GCTGGAGAACCCATGAGGT |  |
| CHOP | Forward | GGAAACAGAGTGGTCATTCCC | 116 |
|  | Reverse | CTGCTTGAGCCGTTCATTCTC |  |
| Malectin | Forward | CCCGAGAGCGTCATTTGGG | 139 |
|  | Reverse | AACGCAGGATTGGCAGTTTCA |  |
| EIF2AK3 | Forward | ACGATGAGACAGAGTTGCGAC | 80 |
|  | Reverse | ATCCAAGGCAGCAATTCTCCC |  |
| BCL2L11 | Forward | TAAGTTCTGAGTGTGACCGAGA | 96 |
|  | Reverse | GCTCTGTCTGTAGGGAGGTAGG |  |
| EP300 | Forward | AGCCAAGCGGCCTAAACTC | 144 |
|  | Reverse | TCACCACCATTGGTTAGTCCC |  |
| THBS1 | Forward | AGACTCCGCATCGCAAAGG | 157 |
|  | Reverse | TCACCACGTTGTTGTCAAGGG |  |
| OPA1 | Forward | TGTGAGGTCTGCCAGTCTTTA | 141 |
|  | Reverse | TGTCCTTAATTGGGGTCGTTG |  |
| SIRT1 | Forward | TAGCCTTGTCAGATAAGGAAGGA | 160 |
|  | Reverse | ACAGCTTCACAGTCAACTTTGT |  |
| Actin | Forward | CTGGAACGGTGAAGGTGACA | 140 |
|  | Reverse | AAGGGACTTCCTGTAACAACGCA |  |

Supplementary Figure S10. Gating strategy for apoptosis analysis by flow cytometry. (A) Selection of intact cells using FSC-A/SSC-A. (B) Exclusion of doublets by FSC-H/FSC-A. (C) Unstained and single-color controls were used to establish fluorescence background and compensation settings. Apoptotic populations were quantified by double staining with Annexin V/PI: Annexin V⁺/PI⁻ (early apoptosis) and Annexin V⁺/PI⁺ (late apoptosis/necrosis). Data are representative of the OE-NC + HG group.
