# Supplementary material for: Malectin Alleviates Endoplasmic Reticulum Stress in Gestational Diabetes Mellitus via Glycoprotein Quality Control Mechanisms
Source: Adv Sci (Weinh). 2026 May 25;13(46):e08901. doi: 10.1002/advs.202508901 (PMC13335976; doi:10.1002/advs.202508901)
Supplement: Supplementary file 2 [file ADVS-13-e08901-s001.zip › advs75803-sup-0002-Data/Supplementary File 2 (Source Data).docx]

**Source data**

Malectin Alleviates Endoplasmic Reticulum Stress in Gestational Diabetes Mellitus via Glycoprotein Quality Control Mechanisms

Jiahui Zhu, Yumeng Zhang, Ye Wang, Xiaoyu Zhu, Ailin Yuan, Weijing Yin, Huangmin Yu, Xuemin Pang, Yufeng He, Yuchen Wang, Tong Wang, Yong Li*, Yunlong Si*.

**Unprocessed western blots**

**Figure 1.**

**Figure 2.**

**Figure 3.**

**Figure 4.**

**Figure 8.**

**Figure 9.**

**Figure 10.**

**1.** **Post-electrophoresis gel imaging.** The Blue-Native PAGE gel was imaged immediately after electrophoresis to document the initial separation pattern.

**2. PVDF membrane alignment.** A PVDF membrane was cut to match the exact dimensions of the gel. The membrane was overlaid on the gel, and an image was captured to record their precise alignment.

**3. Marker lane processing for Coomassie staining.** The lane containing the native protein marker was carefully excised vertically from the gel. This excised marker lane strip was subsequently stained with Coomassie Brilliant Blue to visualize the molecular weight standards.

**4. Wet transfer and post-transfer imaging.** The remaining gel section (containing the protein samples) was subjected to wet electroblotting onto the pre-aligned PVDF membrane. Upon completion of the transfer, an image of the gel and membrane assembly was taken.

**5. Membrane separation and imaging.** Following the transfer, the PVDF membrane was carefully separated from the gel. Both the membrane and the post-transfer gel were imaged separately.

**6. Image composite assembly.** The individual images of the PVDF membrane, the post-transfer gel, and the de-stained marker lane were digitally merged to generate a composite image for accurate band alignment and molecular weight assignment.

**Figure 11.**

**Figure S2.**

**Figure S3.**

**Figure S8.**
